# FLI1 is associated with regulation of DNA methylation and megakaryocytic differentiation in FPDMM caused by a RUNX1 transactivation domain mutation

**DOI:** 10.1038/s41598-024-64829-4

**Published:** 2024-06-18

**Authors:** Yuki Tanaka, Yuri Nakanishi, Erina Furuhata, Ken-ichi Nakada, Rino Maruyama, Harukazu Suzuki, Takahiro Suzuki

**Affiliations:** 1 Laboratory for Cellular Function Conversion Technology, RIKEN Center for Integrative Medical Sciences (IMS), RIKEN Yokohama Campus, 1-7-22 Suehiro-Cho, Tsurumi-Ku, Yokohama City, Kanagawa 230-0045 Japan; 2 Graduate School of Medical Life Science, Yokohama City University, 1-7-29 Suehiro-Cho, Tsurumi-Ku, Yokohama City, Kanagawa 230-0045 Japan; 3 Department of Obstetrics & Gynecology, Juntendo University Faculty of Medicine, 2-1-1 Hongo, Bunkyo-Ku, Tokyo, 113-8421 Japan

**Keywords:** DNA methylation analysis, Familial platelet disorder with associated myeloid malignancies, *FLI1*, DNA demethylation, Megakaryocytic differentiation, Molecular biology, Epigenomics

## Abstract

Familial platelet disorder with associated myeloid malignancies (FPDMM) is an autosomal dominant disease caused by heterozygous germline mutations in *RUNX1*. It is characterized by thrombocytopenia, platelet dysfunction, and a predisposition to hematological malignancies. Although FPDMM is a precursor for diseases involving abnormal DNA methylation, the DNA methylation status in FPDMM remains unknown, largely due to a lack of animal models and challenges in obtaining patient-derived samples. Here, using genome editing techniques, we established two lines of human induced pluripotent stem cells (iPSCs) with different FPDMM-mimicking heterozygous *RUNX1* mutations. These iPSCs showed defective differentiation of hematopoietic progenitor cells (HPCs) and megakaryocytes (Mks), consistent with FPDMM. The FPDMM-mimicking HPCs showed DNA methylation patterns distinct from those of wild-type HPCs, with hypermethylated regions showing the enrichment of ETS transcription factor (TF) motifs. We found that the expression of *FLI1*, an ETS family member, was significantly downregulated in FPDMM-mimicking HPCs with a RUNX1 transactivation domain (TAD) mutation. We demonstrated that FLI1 promoted binding-site-directed DNA demethylation, and that overexpression of *FLI1* restored their megakaryocytic differentiation efficiency and hypermethylation status. These findings suggest that FLI1 plays a crucial role in regulating DNA methylation and correcting defective megakaryocytic differentiation in FPDMM-mimicking HPCs with a RUNX1 TAD mutation.

## Introduction

Familial platelet disorder with associated myeloid malignancies (FPDMM) is an autosomal dominant blood disease characterized by mild-to-moderate thrombocytopenia and defective platelet function. Patients with FPDMM not only bruise and bleed easily, but are also prone to hematological malignancies^1,2^. These patients have a 20–50% life-time risk of myeloid malignancies, such as myelodysplastic syndrome (MDS) and acute myeloid leukemia (AML)^3,4^, indicating FPDMM as precancerous state. FPDMM is caused by heterozygous germline mutations in the RUNX family transcription factor 1 (*RUNX1*) gene. It encodes a transcription factor (TF) and its knockout blocks hematopoietic stem cell formation in vivo and in vitro, defined as a master regulator of definitive hematopoiesis^5–7^. To date, more than 100 different germline *RUNX1* alterations, including missense, nonsense, and frameshift mutations, have been reported across more than 130 epidemiologically identified families^3^.

Unlike humans, animal models such as zebrafish and mice with heterozygous germline *RUNX1* mutations do not have platelet disorders or develop AML^8,9^. Therefore, instead of using animal models, induced pluripotent stem cells (iPSCs) derived from patients with FPDMM have been established as in vitro models^10–12^. In vitro hematopoietic differentiation assays of patient-derived iPSCs showed defects in hematopoietic lineages, particularly in hematopoietic progenitor cell (HPC) emergence and megakaryocyte (Mk) differentiation, indicating the usefulness of these cells as in vitro FPDMM models. However, families with FPDMM are rare worldwide, limiting the acquisition of patient samples. Additionally, the use of patient-derived iPSCs hampers the exploration of the commonality of the molecular signature, due to different genomic backgrounds, and also raises ethical concerns. Therefore, the molecular mechanisms underlying FPDMM progression and the characteristics of cells in FPDMM remain poorly understood.

Previous studies and recent high-throughput sequencing analyses have indicated that aberrant DNA methylation, caused by mutations in DNA methylation-associated genes and TF-encoding genes, is involved in the development of hematological malignancies, such as MDS and AML^13–15^. DNA methylation is a pivotal epigenetic modification. Methylation profiles determine gene expression dynamics during cellular development, differentiation, and maturation^16,17^. We previously found that many TFs, including RUNX1, play a role in regulating DNA demethylation site-specificity^18–21^. However, the aberrant DNA methylation landscape in FPDMM remains unelucidated.

Here, we established two human iPSC lines harboring FPDMM-mimicking *RUNX1* heterozygous (*RUNX1*^+/−^) mutations, generated by means of CRISPR–Cas9-based knockin. We found that these iPSC line-derived HPCs showed a distinct DNA methylation status, with ETS family TF-binding motif-enrichment in hypermethylated regions. We also identified Fli-1 proto-oncogene (FLI1) as a putative causative factor of differential DNA methylation and defective Mk differentiation in FPDMM-mimicking HPCs, particularly in the presence of a mutation in the transactivation domain (TAD) of RUNX1. Our findings provide novel insights into the regulatory mechanisms that shape the DNA methylation landscape and induce Mk differentiation.

## Results

### Establishing human iPSC lines with FPDMM-mimicking *RUNX1* heterozygous mutations

**Figure 1:**
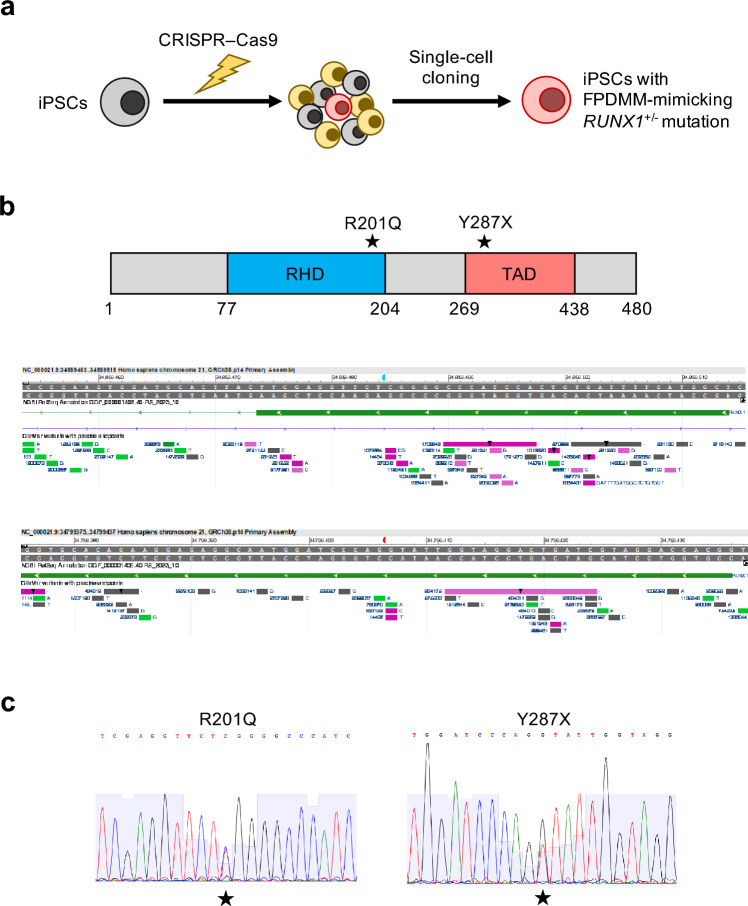
Establishment of FPDMM-mimicking human iPSCs. (**a**) Experimental flow of the approach. A CRISPR–Cas9 complex targeting the specific genomic region was transduced with donor DNA into human iPSCs using electroporation. Single-cell cloning was performed, and the genomic sequence around the targeted region was determined using capillary sequencing and TA cloning. Clones that carried the targeted FPDMM-mimicking *RUNX1* heterozygous mutations were selected and expanded for subsequent experiments. (**b**) (Top) Schematic representation of the two RUNX1 mutations selected in this study. Stars (★) represent the targeted heterozygous mutation sites in RUNX1c (NM_001754.4, 480 amino acid residues). RHD: RUNT homology domain, TAD: transactivation domain. (Bottom) Screenshots of the NCBI Variation Viewer, a browser for genomic variations listed in the publicly available ClinVar database. Highlighted positions represent the nucleotide positions of R201Q (blue) and Y287X (red), respectively, as in the complementary DNA of the canonical transcript (NM_001754.4). (**c**) Bulk sequence traces of RUNX1^WT/R201Q^ (left) and RUNX1^WT/Y287X^ (right) iPSC clones around each targeted region. Stars (★) represent the targeted heterozygous mutation sites.

To establish human iPSC lines with FPDMM-mimicking *RUNX1*^+/−^ mutations, we performed CRISPR–Cas9-mediated genome editing of *RUNX1*, followed by single-cell cloning of the resulting genome-edited cells^22–24^ (Fig. 1a). We introduced two distinct RUNX1 mutations: R201Q and Y287X^3^ (Fig. 1b, top). R201Q (also known as R174Q depending on the RUNX1 isoform) is characterized by a C-to-T transversion in exon 5 that creates a single missense codon in the RUNT homology domain (RHD) that affects RUNX1 functions, such as dimerization with core-binding factor CBFβ, protein stabilization, and DNA-binding, in all three main RUNX1 isoforms, RUNX1a, b, and c^25–29^ (Fig. 1b, bottom). Y287X (also reported as Y260X) is characterized by a G-to-T transversion in exon 7b, which creates a premature stop codon in the TAD and affects the functions of RUNX1b and RUNX1c, but not that of RUNX1a^26,27^.

We confirmed the successful introduction of the *RUNX1* mutations using a T7 endonuclease 1 (T7E1) assay (Supplementary Fig. 1a). We next isolated 37 and 24 single-cell clones from the R201Q-targeted and Y287X-targeted heterogeneous cell populations, respectively. Among these, 2 of the 37 and 2 of the 24 clones showed the RUNX1^WT/R201Q^ (RUNX1 wild-type and R201Q) and RUNX1^WT/Y287X^ (RUNX1 wild-type and Y287X) heterozygous point mutations, respectively (Fig. 1c).

To confirm whether these mutations were monoallelic, we performed TA cloning^30^ of the mutated regions followed by capillary sequencing. Of the 10 *Escherichia coli* clones tested for each mutation, five had the C-to-T transversion representing the R201Q mutation, whereas the other five did not. Additionally, five had the G-to-T transversion representing the Y287X mutation, whereas the other five did not. Furthermore, whole-exome sequencing (WES) analysis confirmed the absence of meaningful off-target mutations in iPSCs with FPDMM-mimicking mutations (Supplementary Fig. 1b,c). Although we identified total eight putative single nucleotide variants (SNVs) at protein-coding regions in two cell lines, of which the expression of genes associated with three of these SNVs showed an upregulation tendency in HPCs compared with iPSCs, none of these genes appeared to be related to FPDMM symptoms or megakaryopoiesis^31–35^, suggesting that the effect of these variants on cellular function, particularly megakaryopoiesis, was insignificant (Supplementary Fig. 1d–f). These results indicated that homologous recombination-mediated heterozygous knockin was accurately introduced at the targeted *RUNX1* locus without significant off-target mutations.

### Human iPSCs with FPDMM-mimicking *RUNX1* heterozygous mutations showed impaired hematopoietic and megakaryocytic differentiation in vitro

**Figure 2:**
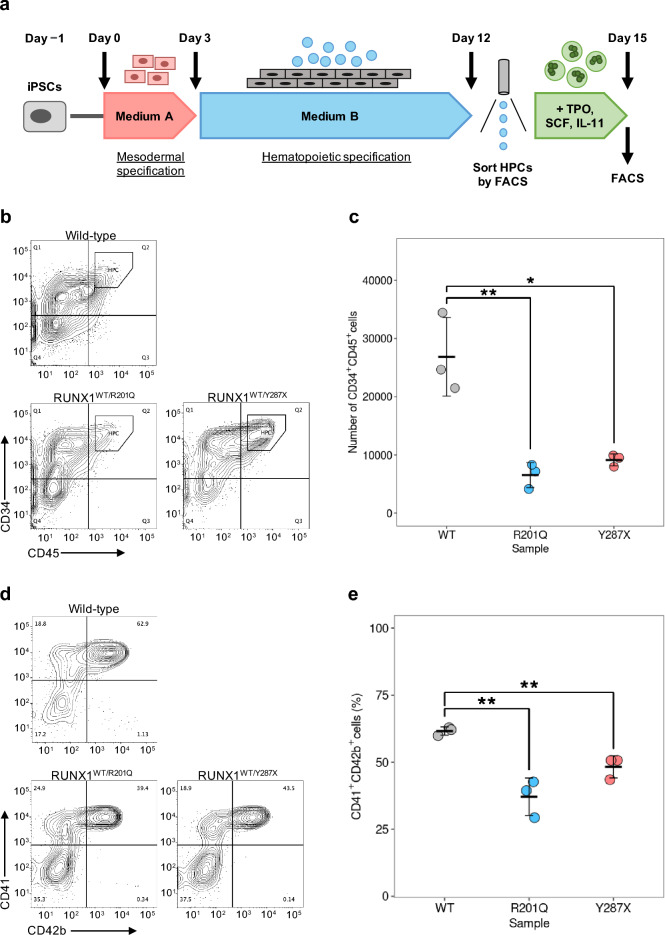
Evaluation of in vitro megakaryocytic differentiation efficiencies of FPDMM-mimicking iPSCs. (**a**) Schematic representation of a megakaryocytic differentiation method starting with iPSCs and ending with Mks via HPCs. (**b**) Representative plots of flow cytometric analysis of CD34^+^CD45^+^ HPCs per wild-type (upper left), RUNX1^WT/R201Q^ (lower left), and RUNX1^WT/Y287X^ iPSC samples (lower right). (**c**) Average absolute numbers of CD34^+^CD45^+^ HPCs in wild-type (WT, gray), RUNX1^WT/R201Q^ (R201Q, blue), and RUNX1^WT/Y287X^ (Y287X, red) iPSC samples per well of a 24-well plate. Data are presented as the mean ± standard deviation (SD) of three biological replicates. Asterisks denote significant difference: *P < 0.05 and **P < 0.01. (**d**) Representative plots of flow cytometric analysis of CD41^+^CD42b^+^ Mks per 20,000 of wild-type (upper left), RUNX1^WT/R201Q^ (lower left), and RUNX1^WT/Y287X^ (lower right) HPCs. (**e**) Percentages of CD41^+^CD42b^+^ Mks per 20,000 wild-type (WT, gray), RUNX1^WT/R201Q^ (R201Q, blue), and RUNX1^WT/Y287X^ (Y287X, red) HPCs. Data are presented as the mean ± SD of three biological replicates. Asterisks denote significant difference: **P < 0.01.

To evaluate the differentiation efficiencies of the human iPSCs with FPDMM-mimicking *RUNX1*^+/−^ mutations into hematopoietic lineage cells, we first induced the differentiation of the *RUNX1*^+/−^ iPSCs into HPCs (Fig. 2a and Supplementary Fig. 2a). Suspended cells were harvested on Day 12 and sorted according to the expression of CD34 and CD45 (a hematopoietic stem/progenitor cell marker and a pan-leukocyte marker, respectively) using flow cytometer (Fig. 2b). We found that the number of HPCs differentiated from RUNX1^WT/R201Q^ and RUNX1^WT/Y287X^ iPSCs was significantly reduced, the output was 76% (P = 0.008, Student’s *t*-test) and 66% (P = 0.042, Welch’s *t*-test) lower, respectively, compared to wild-type iPSCs (Fig. 2c and Supplementary Fig. 2b). Additionally, preliminary single-cell RNA sequencing confirmed a potential bias in the cell state underlying FPDMM-mimicking CD34^+^CD45^+^ HPCs, characterized by a decrease in megakaryoid lineage and early hematopoietic progenitors, while increasing the erythroid lineage^36,37^ (Supplementary Fig. 2c–e).

Next, we induced Mk differentiation in *RUNX1*^+/−^ HPCs (Fig. 2d and Supplementary Fig. 2f.). The proportions of CD41^+^CD42b^+^ Mks differentiated from RUNX1^WT/R201Q^ and RUNX1^WT/Y287X^ HPCs were significantly reduced, with frequencies 40% (P = 0.004, Student’s *t*-test) and 32% (P = 0.007, Student’s *t*-test) lower than those of wild-type HPCs (Fig. 2e and Supplementary Fig. 2g).

These findings indicate that *RUNX1* heterozygous mutation impairs the hematopoietic and megakaryocytic differentiation potential of iPSCs.

### Differentially methylated CpGs in FPDMM-mimicking HPCs

**Figure 3:**
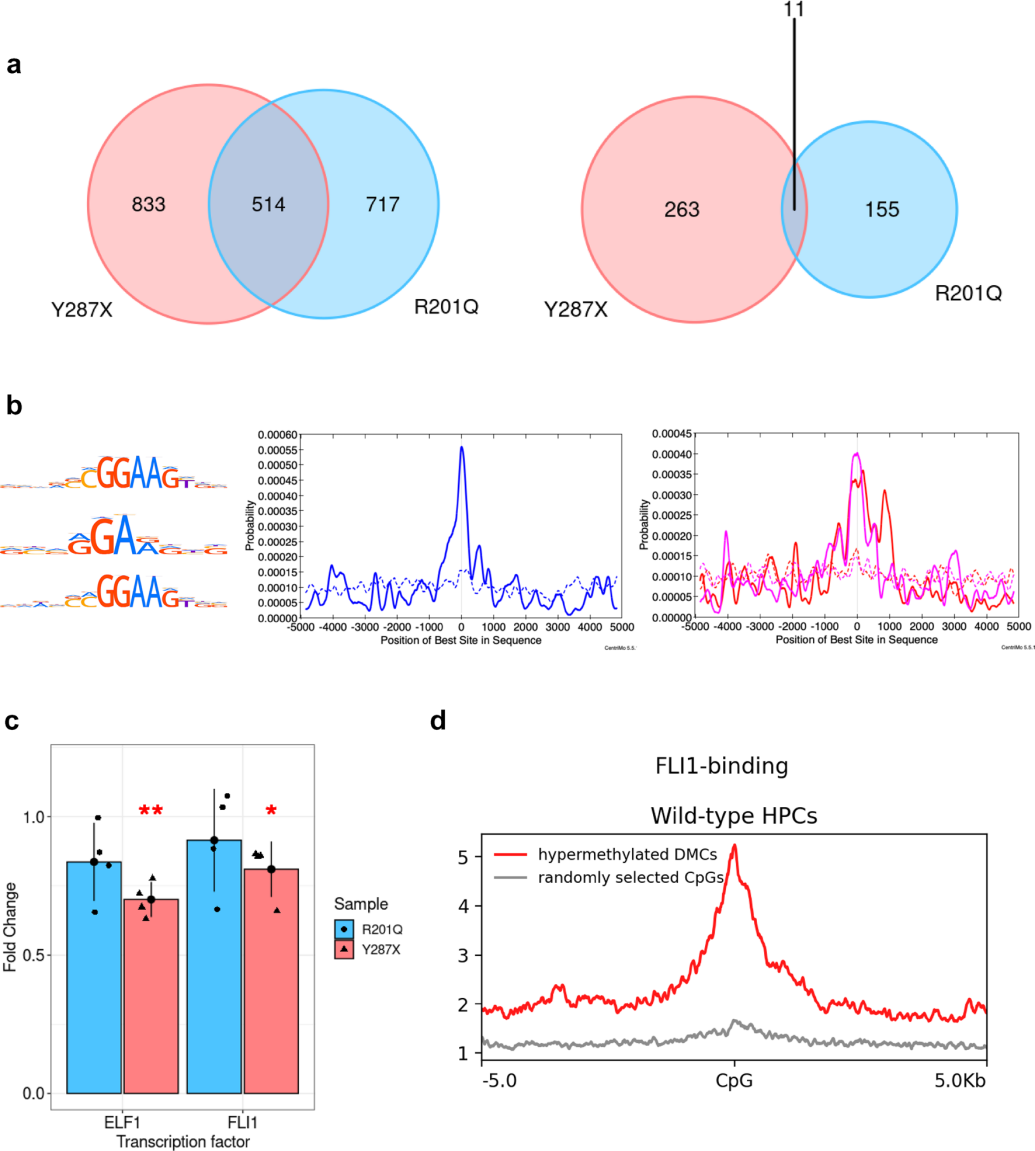
DNA methylation analysis of FPDMM-mimicking HPCs. (**a**) Overlaps of hypermethylated (left) and hypomethylated (right) CpGs between RUNX1^WT/R201Q^ (blue) and RUNX1^WT/Y287X^ (red) HPCs. (**b**) (Left) The known HOCOMOCO v11 binding motif for GABPA (top), FEV (middle), and ELF1 (bottom). (Middle and right) Distribution of ETS family TF-binding motif-enrichment. The *X*- and *Y*-axes show the distance from DMC (bp) and probability of TF-binding motifs, respectively. Solid lines are probabilities at ± 5 kb for hypermethylated DMCs in RUNX1^WT/R201Q^ (middle) and RUNX1^WT/Y287X^ (right) HPCs, and dashed lines are probabilities at ± 5 kb from randomly selected CpGs. Data that are significantly enriched (*E*-value of the Fisher’s exact test < 0.05) and ranked in the top-20 of Fisher *E*-value rankings are presented. Blue: GABPA-binding motif, red: FEV-binding motif, and pink: ELF1-binding motif. (**c**) Expression of *ELF1* (left) and *FLI1* (right) in RUNX1^WT/R201Q^ (blue) and RUNX1^WT/Y287X^ (red) HPCs compared to wild-type cells, confirmed using qRT-PCR. The *X*-axis shows the target genes, and the *Y*-axis represents the fold-change. Data are presented as the mean ± SD of four biological replicates. Asterisks denote significant difference: *P < 0.05 and **P < 0.01. (**d**) Distribution of enrichment for FLI1-binding sites, as determined by CUT&RUN sequencing for FLI1 in wild-type HPCs, showing the regions within ± 5 kb of the hypermethylated DMCs in RUNX1^WT/Y287X^ HPCs (red line). Gray line represents FLI1-binding site-enrichment at regions around randomly selected CpGs.

We performed single-base resolution methylome analyses to characterize the global DNA methylation status of *RUNX1*^+/−^ HPCs. By filtering out read depths of less than 10×, we detected 3,844,825, 5,610,028, and 6,329,803 cytosine-guanine dinucleotides (CpGs) in RUNX1^WT/R201Q^, RUNX1^WT/Y287X^, and wild-type HPCs, respectively. Of these, 781,298 CpGs were present in the intersection among all samples. We identified 1231 hypermethylated and 166 hypomethylated CpGs in RUNX1^WT/R201Q^ HPCs and 1347 hypermethylated and 274 hypomethylated CpGs in RUNX1^WT/Y287X^ HPCs compared with wild-type HPCs using a logistic regression test, indicating a clear bias towards hypermethylation. Of these, 514 hypermethylated and 11 hypomethylated CpGs were altered in common between RUNX1^WT/R201Q^ and RUNX1^WT/Y287X^ HPCs, with a greater overlap observed among the hypermethylated CpGs (Fig. 3a).

We previously reported that TF-binding motif-enrichment analysis of differentially methylated regions predicted DNA methylation-regulating TFs^18–21^. To identify the putative TFs responsible for differential DNA methylation, we performed a TF-binding motif-enrichment analysis using CentriMo^38^. Interestingly, we noticed that binding motifs for ETS family TFs, such as GABPA, FEV, and ELF1 (Fig. 3b, left), were significantly enriched in the vicinity of hypermethylated differentially methylated CpGs (DMCs) in both RUNX1^WT/R201Q^ and RUNX1^WT/Y287X^ HPCs (Fisher *E*-value = 1.3e-29 [GABPA]; Fig. 3b, middle; Fisher *E*-value = 6.6e-34 and 7.4e-22 [FEV and ELF1, respectively]; Fig. 3b, right). ETS family TF motifs were also enriched in the vicinity of the commonly hypermethylated DMCs between RUNX1^WT/R201Q^ and RUNX1^WT/Y287X^ HPCs (Fisher *E*-value = 1.4e-18, 8.5e-17, 5.3e-16, 3.9e-13, and 4.1e-13 [FEV, ELF1, GABPA, ELF2, and ELK1, respectively]; Supplementary Fig. 3a) and RUNX1^WT/Y287X^ HPC-specific hypermethylated DMCs (Fisher *E*-value = 1.6e-16 [FEV]; Supplementary Fig. 3b), but not RUNX1^WT/R201Q^ HPC-specific hypermethylated DMCs. We did not detect any significantly enriched TF-binding motifs in the vicinity of hypomethylated DMCs in either RUNX1^WT/R201Q^ or RUNX1^WT/Y287X^ HPCs. Notably, the RUNX1-binding motif was not significantly enriched in the vicinity of hypo- or hypermethylated DMCs (Supplementary Fig. 3c).

These results suggested that, despite the slight differences between *RUNX1*^+/−^ mutations, ETS family TFs are significantly involved in establishing the differential hypermethylation profiles in FPDMM-mimicking HPCs.

### Identification of ETS family TFs associated with differential hypermethylation in FPDMM-mimicking HPCs

Because the ETS family consists of 28 protein-coding genes in humans^39^ that share the same or similar binding motifs (ETS core sequence “GGAA”), the TF-binding motif-enrichment analysis often included false positives and did not specifically identify corresponding ETS family TFs. Therefore, to identify putative ETS family TFs associated with differential hypermethylation in FPDMM-mimicking HPCs, we examined the expression of ETS family TF genes using quantitative reverse transcription-polymerase chain reaction (qRT-PCR). We detected a statistically significant decrease in the expression of *ELF1* and *FLI1* in RUNX1^WT/Y287X^ HPCs (*P* = 0.003 and *P* = 0.032, respectively; Welch’s *t*-test; Fig. 3c and Supplementary Fig. 3d) compared with that in wild-type cells. In addition, we observed a downregulation tendency in RUNX1^WT/R201Q^ HPCs, although without statistical significance (*P* = 0.10 and *P* = 0.43, respectively; Welch’s *t*-test). We further confirmed that *FLI1* was consistently downregulated in cells hematopoietically differentiated from iPSCs derived from patients with FPDMM bearing the RUNX1^WT/Y287X^ mutation (GSE54295) (Supplementary Fig. 3e). Similarly, *ELF1* tended to be downregulated, albeit with some variability.

Meanwhile, the ETV6-binding motif (SAGGAAR), similar to the FLI1-binding motif (SRRGGMAGGAAGGRRRGR), was significantly enriched in the vicinity of hypermethylated DMCs in RUNX1^WT/Y287X^ HPCs (Fisher *E*-value = 1.9e-2; Supplementary Fig. 3f). However, cleavage under targets and release using nuclease (CUT&RUN) sequencing for FLI1 showed that FLI1-binding was notably enriched in the vicinity of hypermethylated DMCs in wild-type HPCs (Fig. 3d). These findings indicate that FLI1 is associated with differential hypermethylation in RUNX1^WT/Y287X^ HPCs.

### Ectopic overexpression of FLI1 induced binding site-directed DNA demethylation across the genome

**Figure 4:**
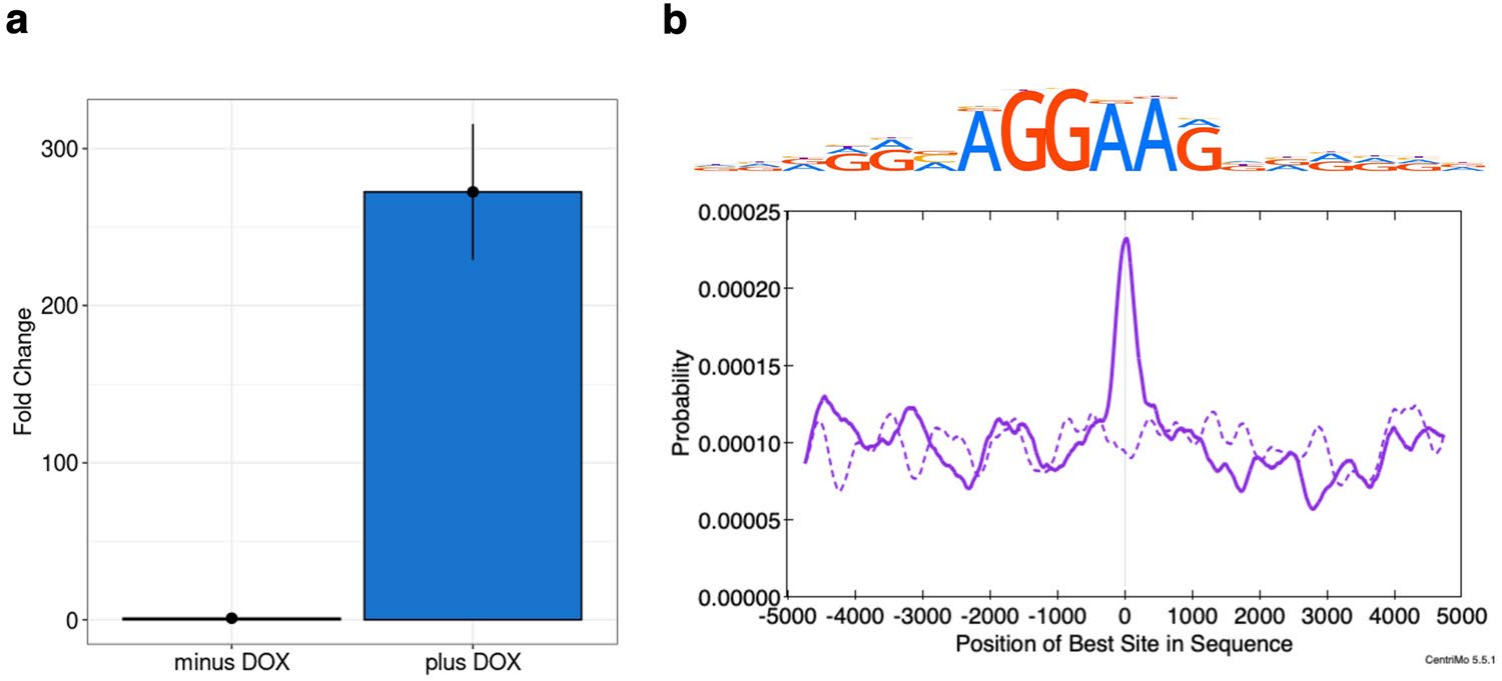
Identification of site-specificity of DNA demethylation in FLI1. (**a**) Confirmation of the DOX-inducible expression of *FLI1* in DOX-treated iPSCs (plus DOX) compared with untreated iPSCs (minus DOX) by qRT-PCR. (**b**) (Top) The known HOCOMOCO v11 binding motif for FLI1. S: G/C, R: A/G, and M: A/C. (Bottom) Distribution of FLI1-binding motif-enrichment. The solid line represents the probability at ± 5 kb from demethylated CpGs in *FLI1*-overexpressing iPSCs, and the dashed line represents the probability at ± 5 kb from randomly selected CpGs.

To validate whether FLI1 induces DNA demethylation at its binding sites in vitro, we overexpressed FLI1 and performed methylome analysis. We established an iPSC line harboring doxycycline (DOX)-inducible *FLI1* using a lentivirus vector in which *FLI1* was ectopically overexpressed upon DOX treatment (272.4-fold increase by highly concentrated DOX in this case; Fig. 4a). Comparing the methylomes of *FLI1*-overexpressing and non-overexpressing iPSCs, we identified 1620 demethylated and 2919 methylated CpGs at a differential methylation level of > 80%. TF-binding motif-enrichment analysis showed that the FLI1-binding motif (SRRGGMAGGAAGGRRRGR) was significantly enriched in the immediate vicinity of demethylated CpGs (Fisher *E*-value = 5.4e-3; Fig. 4b). Based on a substantial upregulation of *FLI1* expression upon DOX treatment in iPSCs, these results indicate that FLI1 induces DNA demethylation in a binding site-directed manner. Meanwhile, the overlap between these demethylated CpGs and the hypermethylated CpGs in FPDMM-mimicking HPCs was 1 for RUNX1^WT/R201Q^ HPCs and 0 for RUNX1^WT/Y287X^ HPCs, showing little overlap (Supplementary Fig. 4).

### *FLI1* downregulation repressed megakaryocytic differentiation

**Figure 5:**
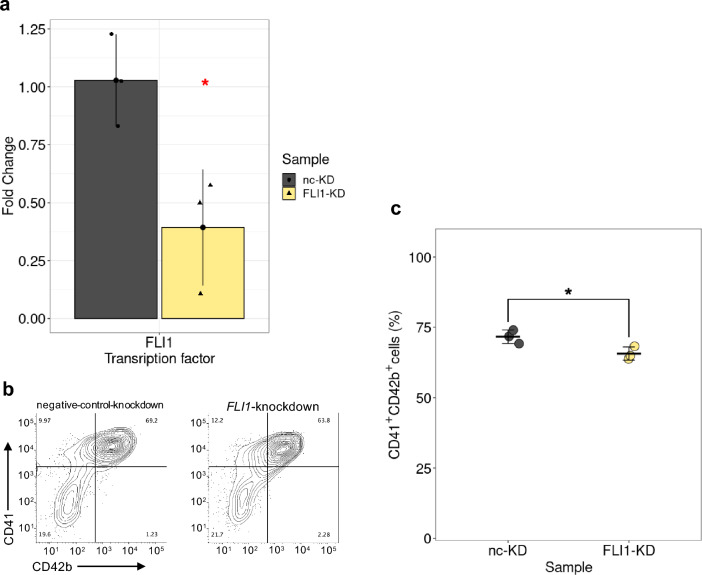
Effect of *FLI1* downregulation on megakaryocytic differentiation. (**a**) Confirmation of the expression of *FLI1* in negative-control-knockdown (nc-KD, gray) and *FLI1*-knockdown (FLI1-KD, yellow) HPCs compared with that in wild-type HPCs using qRT-PCR. The *X*-axis indicates the target genes, and the *Y*-axis indicates the fold-change. Data are presented as the mean ± SD of three biological replicates. Asterisks denote significant difference: *P < 0.05. (**b**) Representative plot for flow cytometric analysis of CD41^+^CD42b^+^ Mks per 20,000 negative-control-knockdown and *FLI1*-knockdown HPCs. (**c**) Percentages of CD41^+^CD42b^+^ Mks per 20,000 negative-control-knockdown (nc-KD, gray) and *FLI1*-knockdown (FLI1-KD, yellow) HPCs. Data are presented as the mean ± SD of three biological replicates. Asterisks denote significant difference: *P < 0.05.

To determine the impact of FLI1 on megakaryopoiesis, we performed constant knockdown of *FLI1* using short hairpin RNA in the entire differentiation process from iPSCs to Mks (Fig. 5a). We found that the average numbers of CD34^+^CD45^+^ HPCs on Day 12 were not significantly decreased in *FLI1*-knockdown cells relative to that in negative-control cells (Supplementary Fig. 5a,b). However, the ratios of CD41^+^CD42b^+^ Mks were significantly decreased in *FLI1*-knockdown HPCs by 3 days after the Mk differentiation onset (Day 15) (*P* = 0.036, Student’s *t*-test) compared with that in the negative-control cells, suggesting the contribution of FLI1 to Mk differentiation (Fig. 5b,c).

### *FLI1* overexpression rescued megakaryocytic differentiation efficiency

**Figure 6:**
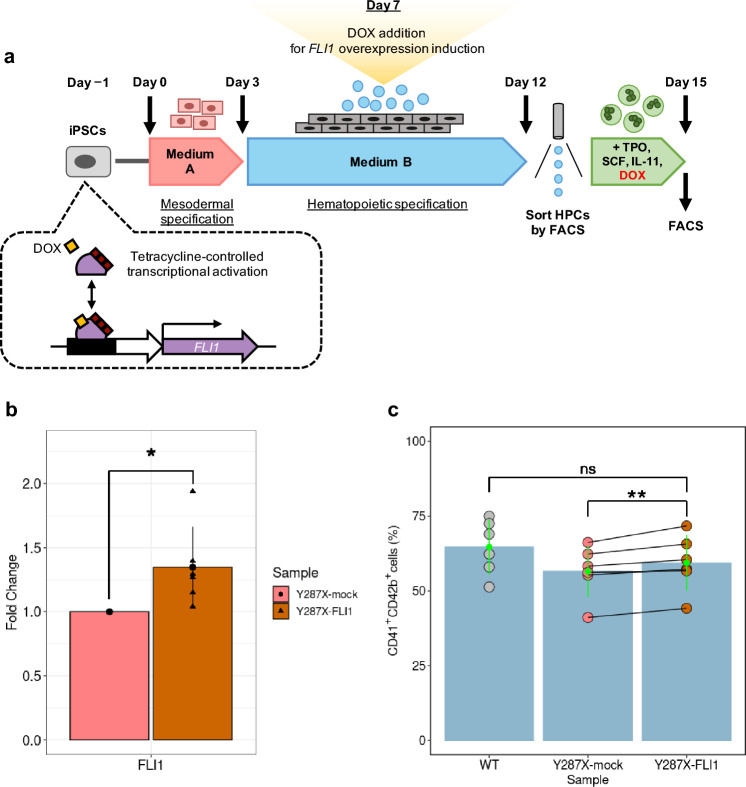
Rescue of deficient megakaryocytic differentiation in FPDMM-mimicking HPCs by *FLI1* overexpression. (**a**) Schematic representation of the megakaryocytic differentiation method. FPDMM-mimicking iPSCs were transfected with a lentiviral vector containing the DOX-inducible *FLI1* expression system. (**b**) Confirmation of the expression of *FLI1* in Y287X-mock (red) and Y287X-FLI1 (orange) HPCs using qRT-PCR. Data are presented as the mean ± SD of six biological replicates. The asterisk denotes significant difference: *P < 0.05. (**c**) Percentages of CD41^+^CD42b^+^ Mks per 20,000 wild-type (WT, gray), Y287X-mock (red), and Y287X-FLI1 (orange) HPCs. Data are presented as the mean ± SD of six biological replicates. The asterisk denotes significant difference: **P < 0.01 and ns, not significant.

To evaluate whether FLI1 overexpression improved deficient Mk differentiation in RUNX1^WT/Y287X^ cells, we established a RUNX1^WT/Y287X^ iPSC line expressing *FLI1* upon DOX treatment and mock control cell line transfected with an empty lentivirus vector (Y287X-FLI1 and Y287X-mock, respectively; Fig. 6a). After adding low-concentrated DOX from Day 7 of hematopoietic differentiation, we evaluated the expression of *FLI1* in HPCs, HPC emergence on Day 12, and Mk differentiation efficiency on Day 15. qRT-PCR results revealed an average 1.3-fold increase in the expression of *FLI1* (*P* = 0.043, paired *t*-test) in Y287X-FLI1 HPCs compared with Y287X-mock HPCs (Fig. 6b).

Although HPC emergence was comparable in Y287X-FLI1 and Y287X-mock cells (Supplementary Fig. 6a,c), Mk differentiation efficiency was significantly improved by *FLI1* overexpression in Y287X-FLI1 HPCs compared with that in Y287X-mock cells (*P* = 0.010, paired *t*-test; Fig. 6c; Supplementary Fig. 6b), but not in R201Q-FLI1 HPCs compared with that in R201Q-mock cells (Supplementary Fig. 6d–f). Collectively, these results suggested that FLI1 rescues deficient Mk differentiation caused by heterozygous mutations in the TAD of RUNX1.

### ***FLI1*** overexpression improved differential hypermethylation in RUNX1^WT/Y287X^ HPCs

**Figure 7:**
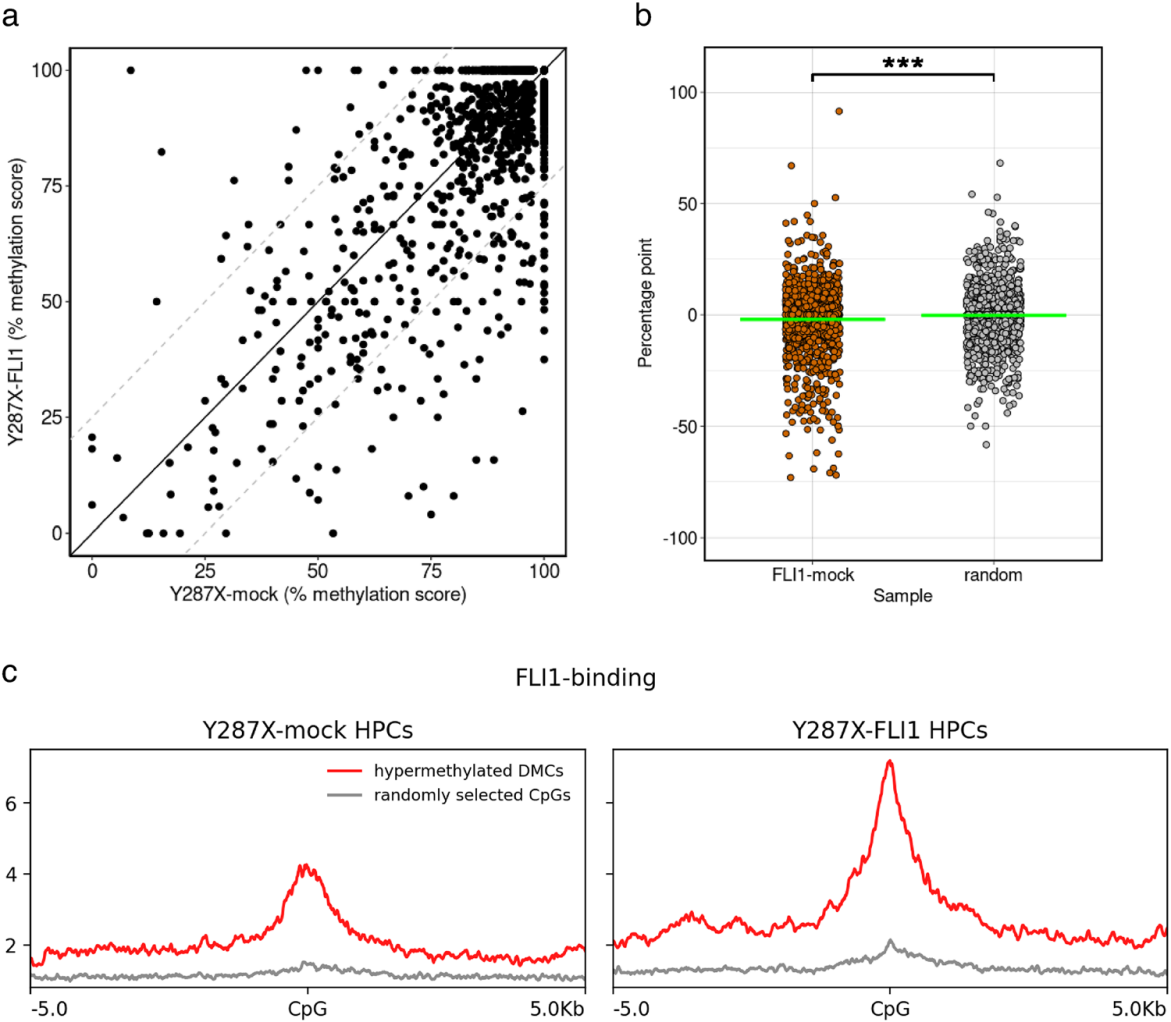
Induction of DNA hypomethylation in FPDMM-mimicking HPCs by *FLI1* overexpression. (**a**) Scatter plot showing the percent methylation scores of 1344 CpG sites between Y287X-mock and Y287X-FLI1 HPCs. The *X*- and *Y*-axes indicate percent methylation scores for Y287X-mock and Y287X-FLI1 HPCs, respectively. The solid line represents equal percent methylation scores between samples, and dashed lines represent differences in percent methylation scores of > 25%. (**b**) Percentage point distributions of 1344 CpG sites between Y287X-FLI1 and Y287X-mock HPCs (FLI1-mock, orange) and of the same number of CpG sites randomly selected among CpG sites covered by sequencing (random, gray). Data are presented as the mean ± SD. The asterisk denotes significant difference: ***P < 0.001. (**c**) Distributions of enrichment for FLI1-binding sites, as determined by CUT&RUN sequencing for FLI1 in Y287X-mock (left) and Y287X-FLI1 HPCs (right), showing the regions within ± 5 kb of the hypermethylated DMCs in RUNX1^WT/Y287X^ HPCs (red lines). Gray lines represent FLI1-binding site-enrichments at regions around randomly selected CpGs.

We performed single-base resolution methylome analyses to explore effect of *FLI1* overexpression at the hypermethylated DMCs in RUNX1^WT/Y287X^ HPCs. Comparing the percent methylation scores of these CpGs between Y287X-FLI1 and Y287X-mock HPCs, we identified an overall bias towards hypomethylation in Y287X-FLI1 HPCs (Fig. 7a and Supplementary Table 1). For example, the score of chr1: 111204637–111204638 was decreased by approximately 73% (Supplementary Fig. 7a). The mean percentage point of these CpGs between Y287X-FLI1 and Y287X-mock HPCs (percent methylation score of Y287X-FLI1 HPCs minus that of Y287X-mock HPCs) was significantly lower than that of the same number of randomly selected CpG sites (mean = −2.03 and −0.228 [FLI1-mock and random, respectively]; *P* = 0.0006, Welch’s *t*-test; Fig. 7b; Supplementary Fig. 7b). In addition, the changes in FLI1-binding dynamics for the hypermethylated DMCs between Y287X-mock and Y287X-FLI1 HPCs were confirmed by CUT&RUN sequencing, and these results showed that FLI1-binding surrounding the DMCs was significantly increased in Y287X-FLI1 HPCs compared to Y287X-mock HPCs (Fig. 7c).

Thus, *FLI1* overexpression improved not only the Mk differentiation efficiency, but also the differential hypermethylation in RUNX1^WT/Y287X^ HPCs.

## Discussion

We established human iPSC lines with FPDMM-mimicking *RUNX1* heterozygous mutations using CRISPR–Cas9-based knockin. The iPSCs harboring FPDMM-mimicking *RUNX1*^+/−^ mutations demonstrated impaired differentiation into HPCs and Mks compared with wild-type iPSCs. Furthermore, the FPDMM-mimicking HPCs showed DNA methylation patterns distinct from those of wild-type HPCs, with ETS family TF motifs being enriched in hypermethylated regions. We also revealed the downregulation of *FLI1* in the FPDMM-mimicking HPCs, particularly in the presence of a nonsense mutation in the TAD of RUNX1. In addition, we demonstrated that FLI1 has site-specific DNA demethylation-inducing activity. Knockdown of *FLI1* inhibited Mk differentiation, whereas its overexpression restored Mk differentiation and differential hypermethylation in iPSC-derived HPCs harboring the RUNX1^WT/Y287X^ mutation. These findings indicated that FLI1 plays a pivotal role in regulating DNA demethylation in FPDMM-mimicking HPCs, particularly in the presence of a RUNX1 TAD mutation. As this mutation reflect that causing FPDMM, our results provide insights into one aspect of FPDMM pathology.

Our approach offers the advantage of analyzing cells harboring different mutations against the same genetic background. This allowed us to investigate common molecular signatures caused only by *RUNX1* heterozygous mutations. The R201Q mutation recapitulated in this study is located in RHD, resulting in defective DNA binding^26,28^, whereas the Y287X mutation is a nonsense mutation in TAD, appearing to be a dominant-negative effect^26,27^. Additionally, the R201Q mutation affects all three RUNX1 isoforms, whereas the Y287X mutation does not affect RUNX1a, a RUNX1 isoform that plays a role in enhancing the in vitro differentiation of HPCs from human embryonic stem cells and iPSCs^40^. We found that *ELF1* and *FLI1* were differentially downregulated in RUNX1^WT/R201Q^ and RUNX1^WT/Y287X^ HPCs, even though they were causative candidates for differential hypermethylation and defective Mk differentiation. Thus, although the definition of FPDMM is independent of the type or location of the inherited *RUNX1* mutation, our results suggested diverse features related to *RUNX1* mutation position. Indeed, the frequency of MDS/AML development is higher in patients with FPDMM with mutations in RHD of RUNX1 than in those with mutations in TAD^41,42^. Therefore, combining iPSCs and genome editing is advantageous for studying genetic diseases. In a future study, we will establish iPSCs with other types of *RUNX1*^+/−^ mutations to further understand FPDMM pathogenesis.

We found that ETS family TF-binding motifs were enriched in hypermethylated DMCs in both RUNX1^WT/R201Q^ and RUNX1^WT/Y287X^ HPCs. Since the results of TF-binding motif-enrichment analysis are often subject to fluctuations due to the inclusion of false positives, we tested the expression of all ETS family TFs and found that *ELF1* and *FLI1* were significantly downregulated in RUNX1^WT/Y287X^ HPCs. Overexpression of FLI1 induced binding-site-directed DNA demethylation in iPSCs and improved impaired Mk differentiation and differential hypermethylation in RUNX1^WT/Y287X^ cells. By analyzing CUT&RUN data for FLI1 between wild-type, Y287X-mock, and Y287X-FLI1 HPCs, we further demonstrated the capability of FLI1 to bind to hypermethylated DMCs and the restoration of this differential hypermethylation through FLI1-binding by overexpression of *FLI1*. Recently, next generation sequencing-based studies reported *FLI1* mutations that disrupt its DNA-binding ability as a cause of FPD^43^, supporting our findings. Although ELF1 was excluded from this study because of its lack of interference with DNA demethylation^20^, it has been reported as a FLI1 target gene^44^. Indeed, the expression patterns of *ELF1* and *FLI1* were correlated in AML cells^45^, whereas no evidence to date suggests that ELF1 is crucial for megakaryopoiesis. Therefore, the observed *ELF1* downregulation may be attributed to the decreased expression of *FLI1*. These findings suggested that FLI1 is a critical causative gene in pathogenesis of FPDMM caused by a RUNX1 TAD mutation, which could serve as a potential drug target.

The reason for the downregulation of the expression of *FLI1* in HPCs by a RUNX1 TAD mutation remains unclear. FLI1 is also known to regulate the expression of RUNX1^44,46^. However, no studies to date have indicated that RUNX1 directly regulates the expression of FLI1. Thus, RUNX1 may indirectly regulate the expression of FLI1.

The specific gene expression regulated by differential DNA methylation identified in FPDMM-mimicking *RUNX1*^+/−^ HPCs in this study remains unclear. It has been reported that gene expression is regulated by CpG methylation marks in proximal promoter regions; however, the positive or negative correlation between DNA methylation and gene expression is not straightforward because not all CpGs significantly influence gene expression based on their methylation status^47^. In addition, differential DNA methylation occurs not only in promoters but also in gene bodies^48^, making it difficult to investigate the functional involvement of DNA methylation, i.e. which gene expressions are regulated by specific DNA methylation patterns. Hi-C sequencing, which elucidates the overall genomic structure of mammalian chromosomes and the biophysical properties of chromatin, can address these complexities.

We previously reported that RUNX1 induces binding-site-directed DNA demethylation^18^. However, the RUNX1-binding motif was not enriched around DMCs in FPDMM-mimicking HPCs harboring *RUNX1*^+/−^ mutations. Therefore, our results suggested that RUNX1^R201^^Q^ and RUNX1^Y287X^ in FPDMM-mimicking HPCs predominantly affect downstream gene regulation, but not DNA demethylation-inducing activity. Alternatively, wild-type RUNX1 expressed from another allele may be sufficient to establish a RUNX1-mediated DNA methylation profile.

None of the ETS family TFs tested were significantly downregulated in RUNX1^WT/R201Q^ HPCs despite the enrichment of ETS family TF motifs in the vicinity of the hypermethylated DMCs. Because ETS family TFs bind to similar motifs and several ETS family TFs tended to be downregulated in RUNX1^WT/R201Q^ HPCs, the hypermethylation observed in RUNX1^WT/R201Q^ HPCs may be due to the combinatorial effect of multiple ETS family TFs. Another possibility is that, because none of the specific TF-binding motifs were enriched in the RUNX1^WT/R201Q^ HPC-specific hypermethylated DMCs, non-TF-mediated DNA methylation regulatory mechanisms may be involved in RUNX1^WT/R201Q^ HPCs, predominantly contributing to Mk differentiation deficiency.

The overexpression of *FLI1* seemed to mitigate several hypermethylated DMCs identified in RUNX1^WT/Y287X^ HPCs, potentially enhancing Mk differentiation. Our previous research, and this study, indicates that FLI1 may form a DNA demethylation machinery complex involving the DNA demethylase TET2, which it recruits to FLI1-binding sites on DNA^18–20^. This mechanism enables overexpressed FLI1 to direct DNA demethylation at specific binding sites and ameliorate hypermethylation in Y287X-FLI1 HPCs. Conversely, *FLI1* overexpression led to further hypermethylation of several CpGs in Y287X-FLI1 HPCs compared to Y287X-mock cells. The cause of this, whether due to qualitative differences in HPCs or the biological effects of *FLI1* overexpression, warrants further exploration. Meanwhile, the mismatch between demethylated CpGs in *FLI1*-overexpressing iPSCs and hypermethylated CpGs in RUNX1^WT/Y287X^ HPCs may stem from cellular differences, suggesting the involvement of cell type-specific cofactors for FLI1-binding to DNA.

FPDMM-mimicking RUNX1^WT/Y287X^ iPSCs exhibited more impaired hematopoietic and megakaryocytic differentiation in vitro than *FLI1*-knockdown cells, despite experiencing less *FLI1* downregulation. Previous research has demonstrated that the formation of HPCs and Mks involves a robust TF-gene regulatory network, including RUNX1 and FLI1^49,50^, suggesting that *FLI1*-knockdown may only partially disrupt this network, particularly in terms of transcriptomic and epigenetic regulation by FLI1. Therefore, reduced expression of *FLI1* alone may not affect differentiation efficiency.

Although FPDMM does not always have a clear phenotype in Mks and platelets^42,43^, we found that *FLI1* overexpression significantly improved Mk differentiation efficiency in FPDMM-mimicking RUNX1^WT/Y287X^ HPCs. Meanwhile, the deficient Mk differentiation in RUNX1^WT/Y287X^ HPCs was not solely due to *FLI1* expression, suggesting that additional mechanisms by TFs other than FLI1 may also affect the overall efficiency. Thus, restoring expression of other TFs along with *FLI1* might be necessary to achieve Mk differentiation efficiency in Y287X-FLI1 HPCs comparable to that of wild-type cells.

In conclusion, we successfully established human iPSC lines bearing heterozygous mutations in *RUNX1* as an FPDMM-mimicking model. This study demonstrated the defective Mk differentiation and differential DNA methylation in FPDMM-mimicking HPCs. Our results suggested that the downregulation of *FLI1* is responsible for differential DNA methylation and Mk differentiation, at least in cells with a heterozygous nonsense mutation in TAD of RUNX1. FPDMM-mimicking iPSCs are a valuable tool for FPDMM research. Because FPDMM is a preleukemic state, these FPDMM-mimicking iPSC lines will also be a useful resource for investigating leukemic transformation.

## Methods

### Cell culture

The 610B1 human iPSC line (HPS0331; RIKEN BRC, Tsukuba, Japan) was cultured on Corning® Matrigel® hESC-Qualified Matrix (Corning Inc., Corning, NY, USA) in mTeSR™ Plus-cGMP medium (STEMCELL Technologies Inc, Vancouver, BC, Canada) and passaged every 6–7 days using ReLeSR™ (STEMCELL Technologies Inc.), following the manufacturer's protocol.

### CRISPR–Cas9 editing and single-cell cloning

Alt-R® CRISPR–Cas9 crRNAs (Integrated DNA Technologies, Coralville, IA, USA) and donor DNA templates are detailed in Supplementary Table 2. CRISPR–Cas9 editing involved assembling and electroporating ribonucleoprotein complexes with the Neon Transfection System (Invitrogen, Waltham, MA, USA) as per the manufacturer’s protocol. As a control, wild-type iPSCs not transfected a non-targeting CRISPR–Cas9 construct were used. Mutation-introduction efficiency was determined using T7E1 assays with an Alt-R® Genome Editing Detection Kit (Integrated DNA Technologies) following the manufacturer’s instructions. The polymerase chain reaction (PCR) primers used are listed in Supplementary Table 3. Single-cell clones generated from CRISPR-edited populations were cultured in mTeSR™ Plus-cGMP medium supplemented with CloneR™ (STEMCELL Technologies Inc.). The PCR primers used in nested PCR to confirm the target genomic sequences are shown in Supplementary Table 4. TArget Clone™-Plus-(TOYOBO, Osaka, Japan) was used to check *RUNX1* alleles according to the manufacturer's protocol, using the primers listed in Supplementary Table 4.

### WES analysis

Genomic DNA was extracted from dissociated cells using a NucleoSpin® Tissue Kit (Macherey–Nagel, Düren, Germany). Azenta Life Sciences Co., Ltd. (Tokyo, Japan) prepared exome libraries, which were sequenced using 150-bp paired-end reads on a DNBSEQ-G400 genome sequencer (MGI, Shenzhen, China). For variant-calling analysis of WES data, Cutadapt^51^ was used to remove the Illumina universal adapter sequence, and PRINSEQ^52^ was used to filter reads based on criteria such as length, quality, and Ns. The data was aligned to the GRCh38/hg38 reference genome using BWA^53–55^. SAMtools^56^ was used to filter the output files. The resulting binary alignment map files were used for SNV and insertion/deletion (indel) discovery. Picard marked over-sequenced reads as duplicates (https://gatk.broadinstitute.org/hc/en-us/articles/360037060272-MarkDuplicates-Picard) and GATK recalibrated base-call errors (https://gatk.broadinstitute.org/hc/en-us/articles/360036898312-BaseRecalibrator). Raw SNVs and indels were detected using the MuTect2^57^, VarScan 2^58^, and Pindel software^59^. Technical artifacts and sequencing errors were evaluated and filtered using the SnpSift software^60^. Putative SNVs and indels identified from different software programs were overlapped using BEDTools^61^. In silico off-target detection of guide RNA sequences was performed using Cas-OFFinder^62^. The PCR primers used in (nested) PCR to confirm the genomic sequences around the putative SNVs validated by WES analysis are shown in Supplementary Table 5.

### Hematopoietic and megakaryocytic differentiation

In vitro hematopoietic differentiation was performed using a STEMdiff™ Hematopoietic Kit (STEMCELL Technologies Inc.) following the manufacturer’s instructions. On Day 12, CD34^+^CD45^+^ HPCs were harvested from the cell culture supernatant containing hematopoietic cells without endothelial lineage cells and sorted using fluorescence-activated cell sorting (FACS). For megakaryocytic differentiation, sorted CD34^+^CD45^+^ HPCs (20,000 cells) were cultured for an additional 3 days in serum-free medium containing 50% Iscove’s Modified Dulbecco’s Medium (Sigma-Aldrich, St. Louis, MO, USA) and 50% Ham’s F12 medium (Sigma-Aldrich) supplemented with 5 mg/mL Bovine Serum Albumin (Sigma-Aldrich), Insulin-Transferrin-Selenium-Ethanolamine (ITS-X; Thermo Fisher Scientific, Waltham, MA, USA), Chemically Defined Lipid Concentrate (Thermo Fisher Scientific), 50 μg/mL L-ascorbic acid (Sigma-Aldrich), 2 mM GlutaMAX™ Supplement (Thermo Fisher Scientific), 437 μM StemSure® Monothioglycerol Solution (FUJIFILM Wako, Osaka, Japan), 50 ng/mL Recombinant Human thrombopoietin (TPO; carrier-free; BioLegend, San Diego, CA, USA), 20 ng/mL Recombinant Human Stem Cell Factor (SCF; Animal-derived-free; FUJIFILM Wako), and 20 ng/mL Recombinant Human Interleukin-11 (IL-11; FUJIFILM Wako).

### Flow cytometry

On Day 12 of hematopoietic differentiation, the supernatants were harvested, and the released cells were stained for CD34 and CD45 (BioLegend). Flow cytometry was performed using a FACSAriaII SORP (BD Biosciences, Franklin Lakes, NJ, USA) with a 100-μm nozzle. On Day 15 of megakaryocytic differentiation, whole cultures were harvested, and cells were stained for Mk markers, CD41 and CD42b (BioLegend), and analyzed as above.

### Enzymatic methyl sequencing analysis

For enzymatic methyl sequencing (EM-seq) analyses, CD34^+^CD45^+^ HPCs were sorted from two independent biological replicates. Genomic DNA was extracted using a NucleoSpin® Tissue XS Kit (Macherey–Nagel), and 39.0–200.0 ng of genomic DNA was used per sample for library construction using the NEBNext® Enzymatic Methyl-seq Kit and Unique Dual Index Primer Pairs (#E7120S; New England Biolabs, Ipswich, MA, USA) following the manufacturer’s instructions. Library size distribution was determined using a High-Sensitivity DNA Kit on a 2100 Bioanalyzer (Agilent Technologies, Santa Clara, CA, USA) and library concentration was quantified using a GenNext® NGS Library Quantification Kit (TOYOBO). These libraries were sequenced using 150-bp paired-end reads on a DNBSEQ-G400 (MGI). The SeqKit^63^, Cutadapt, PRINSEQ++^64^, and FastQC tools were used to reformat, trim, filter, and evaluate the whole-genome sequencing data. The preprocessed FASTQ files were aligned to the GRCh38/hg38 reference genome using Bismark^65^. SAMtools was used to process the output files. Merging of data and evaluation of differences in DNA methylation were performed using the methylKit package^66^, according to the methylKit: User Guide v1.2.4. DMCs were identified using methylation difference > 25% and Q-value < 0.01 as cutoffs. Genomic regions enrichment of annotations tool (GREAT) was used to analyze the functional enrichment significance of DMCs across an entire genome.

### Statistical *t*-test

The Fisher distribution test (*F*-test) was performed as a preliminary assessment to determine whether the subsequent *t*-tests should assume equal or unequal variances. Based on the results, we chose either student *t*-test or Welch's *t*-test.

### TF-binding motif-enrichment analysis

TF-binding motifs within ± 5 kb regions from DMCs were identified using CentriMo^38^, based on hg38 genomic sequence. The motifs were obtained in MEME format from the HOmo sapiens COmprehensive MOdel COllection (HOCOMOCO) v11. Enriched motifs were limited to the top-20 in Fisher *E*-value rankings.

### qRT-PCR

For qRT-PCR analyses, total RNA was extracted using a NucleoSpin® RNA Plus Kit (Macherey–Nagel), reverse-transcribed using a PrimeScript™ RT reagent Kit (Perfect Real Time; Takara Bio, Shiga, Japan), and amplified on a StepOnePlus™ Real-Time PCR System (Applied Biosystems, Waltham, MA, USA) using TB Green® *Premix Ex Taq*™ II (Tli RNaseH Plus; Takara Bio). *GAPDH* mRNA was used as a control for normalization. The qRT-PCR primers used are listed in Supplementary Tables 5 and 6. Gene expression in samples was evaluated using the ΔΔCq method^67^ compared with that in wild-type control.

### CUT&RUN sequencing analysis

CUT&RUN was performed using a CUT&RUN Assay Kit (#86652S; Cell Signaling Technology, Danvers, MA, USA) according to the manufacturer’s instructions. Briefly, the sorted CD34^+^CD45^+^ HPCs were bound to concanavalin A-coated magnetic beads. Primary antibody against human FLI1 (FLI1 (D7N5M) Rabbit mAb; #35980; Cell Signaling Technology) was added and cells were incubated overnight at 4 °C. The next day, the fusion protein pAG-MNase enzyme was added and activated for chromatin digestion, followed by the CUT&RUN fragment extraction. The CUT&RUN sequencing libraries were prepared by DNA Library Prep Kit for Illumina Systems (ChIP-seq, CUT&RUN; Cell Signaling Technology) according to the CUT&RUN protocol. These libraries were sequenced using 50-bp paired-end reads on a NovaSeq X Plus (Illumina, San Diego, CA, USA). The sequence reads were mapped to the GRCh38/hg38 reference genome using Bowtie 2^68^. Duplicated reads, reads mapped to the mitochondrial genome, and reads shorter than 25 bp were removed using SAMtools or command lines in Linux. Sequence coverage was calculated using the bamCoverage function of deepTools2^69^ with the reads per genomic content normalization method.

### Ectopic expression of *FLI1*

The FLI1 open reading frame was subcloned into the pCW57.1 vector using the Gateway LR reaction (Thermo Fisher Scientific), and DOX-inducible FLI1 lentiviral vectors were produced using the LV-MAX Lentiviral Production System (Thermo Fisher Scientific) following the manufacturer’s instructions. These lentiviral vectors were transduced into 610B1 human iPSCs (RIKEN BRC). Transduced cells were selected by culturing with 2 μg/mL puromycin (InvivoGen, San Diego, CA, USA) for two weeks. The overexpression of *FLI1* was induced by adding 500 ng/mL DOX. After seven days, cells overexpressing *FLI1* were harvested and genomic DNA was isolated using a NucleoSpin® Tissue Kit (Macherey–Nagel). EM-seq and qRT-PCR were conducted as described above.

### *FLI1* knockdown

*FLI1* shRNA (sc-35384-SH) and control shRNA (sc-108060) were purchased from Santa Cruz Biotechnology (Dallas, TX, USA). The preparation and transduction of lentiviruses were performed as described above, and knockdown efficiency was confirmed by qRT-PCR.

### *FLI1* rescue

During hematopoietic differentiation, *FLI1* overexpression was induced by replacing half of the culture medium with fresh medium containing 40 ng/mL DOX from Day 7 of hematopoietic differentiation onwards.

### Supplementary Information

Supplementary Information.

Supplementary Table 1.

Supplementary Table 2.

Supplementary Table 3.

Supplementary Table 4.

Supplementary Table 5.

Supplementary Table 6.

## Data Availability

The datasets generated and/or analyzed during the current study are available in the NCBI Gene Expression Omnibus (GEO; http://www.ncbi.nlm.nih.gov/geo/) under accession number GSE245771. R scripts generated for the analysis are available on GitHub (https://github.com/0y3u0k7i/FPD_exome_methyl/).

## References

[1] Heller PG (2005). Low Mpl receptor expression in a pedigree with familial platelet disorder with predisposition to acute myelogenous leukemia and a novel *AML1* mutation. Blood.

[2] Owen CJ (2008). Five new pedigrees with inherited *RUNX1* mutations causing familial platelet disorder with propensity to myeloid malignancy. Blood.

[3] Brown AL (2020). *RUNX1*-mutated families show phenotype heterogeneity and a somatic mutation profile unique to germline predisposed AML. Blood Adv..

[4] Simon L (2020). High frequency of germline *RUNX1* mutations in patients with *RUNX1*-mutated AML. Blood.

[5] Cai Z (2000). Haploinsufficiency of AML1 affects the temporal and spatial generation of hematopoietic stem cells in the mouse embryo. Immunity.

[6] Lacaud G (2002). *Runx1* is essential for hematopoietic commitment at the hemangioblast stage of development in vitro. Blood.

[7] Lacaud G, Kouskoff V, Trumble A, Schwantz S, Keller G (2004). Haploinsufficiency of *Runx1* results in the acceleration of mesodermal development and hemangioblast specification upon in vitro differentiation of ES cells. Blood.

[8] Sood R (2010). Development of multilineage adult hematopoiesis in the zebrafish with a *runx1* truncation mutation. Blood.

[9] Sun W, Downing JR (2004). Haploinsufficiency of *AML1* results in a decrease in the number of LTR-HSCs while simultaneously inducing an increase in more mature progenitors. Blood.

[10] Connelly JP (2014). Targeted correction of *RUNX1* mutation in FPD patient-specific induced pluripotent stem cells rescues megakaryopoietic defects. Blood.

[11] Sakurai M (2014). Impaired hematopoietic differentiation of *RUNX1*-mutated induced pluripotent stem cells derived from FPD/AML patients. Leukemia.

[12] Iizuka H (2015). Targeted gene correction of *RUNX1* in induced pluripotent stem cells derived from familial platelet disorder with propensity to myeloid malignancy restores normal megakaryopoiesis. Exp. Hematol..

[13] Yang X, Wong MPM, Ng RK (2019). Aberrant DNA methylation in acute myeloid leukemia and its clinical implications. Int. J. Mol. Sci..

[14] Blecua P, Martinez-Verbo L, Esteller M (2020). The DNA methylation landscape of hematological malignancies: An update. Mol. Oncol..

[15] Cabezón M (2021). Different methylation signatures at diagnosis in patients with high-risk myelodysplastic syndromes and secondary acute myeloid leukemia predict azacitidine response and longer survival. Clin. Epigenet..

[16] Watt F, Molloy PL (1988). Cytosine methylation prevents binding to DNA of a HeLa cell transcription factor required for optimal expression of the adenovirus major late promoter. Genes Dev..

[17] Smith ZD, Meissner A (2013). DNA methylation: Roles in mammalian development. Nat. Rev. Genet..

[18] Suzuki T (2017). RUNX1 regulates site specificity of DNA demethylation by recruitment of DNA demethylation machineries in hematopoietic cells. Blood Adv..

[19] Suzuki T (2017). A screening system to identify transcription factors that induce binding site-directed DNA demethylation. Epigenet. Chromatin.

[20] Miyajima Y (2022). Prediction of transcription factors associated with DNA demethylation during human cellular development. Chromosome Res..

[21] Suzuki T (2022). GATA6 is predicted to regulate DNA methylation in an in vitro model of human hepatocyte differentiation. Commun. Biol..

[22] Li HL, Gee P, Ishida K, Hotta A (2016). Efficient genomic correction methods in human iPS cells using CRISPR–Cas9 system. Methods.

[23] Liang X, Potter J, Kumar S, Ravinder N, Chesnut JD (2017). Enhanced CRISPR/Cas9-mediated precise genome editing by improved design and delivery of gRNA, Cas9 nuclease, and donor DNA. J. Biotechnol..

[24] Zhang JP (2017). Efficient precise knockin with a double cut HDR donor after CRISPR/Cas9-mediated double-stranded DNA cleavage. Genome Biol..

[25] Bluteau D (2011). Down-regulation of the RUNX1-target gene NR4A3 contributes to hematopoiesis deregulation in familial platelet disorder/acute myelogenous leukemia. Blood.

[26] Okada Y (2013). *RUNX1*, but not its familial platelet disorder mutants, synergistically activates *PF4* gene expression in combination with ETS family proteins. J. Thromb. Haemost..

[27] Sood R, Kamikubo Y, Liu P (2017). Role of RUNX1 in hematological malignancies. Blood.

[28] Kellaway SG (2021). Different mutant RUNX1 oncoproteins program alternate haematopoietic differentiation trajectories. Life Sci. Alliance.

[29] Kellaway SG, Coleman DJL, Cockerill PN, Raghavan M, Bonifer C (2022). Molecular basis of hematological disease caused by inherited or acquired RUNX1 mutations. Exp. Hematol..

[30] Mizuguchi H, Nakatsuji M, Fujiwara S, Takagi M, Imanaka T (1999). Characterization and application to hot start PCR of neutralizing monoclonal antibodies against KOD DNA polymerase. J. Biochem..

[31] Higuchi M (2000). Point mutation in an AMPA receptor gene rescues lethality in mice deficient in the RNA-editing enzyme ADAR2. Nature.

[32] Kubota-Sakashita M, Iwamoto K, Bundo M, Kato T (2014). A role of *ADAR2* and RNA editing of glutamate receptors in mood disorders and schizophrenia. Mol. Brain.

[33] Maroofian R (2021). Biallelic variants in *ADARB1*, encoding a dsRNA-specific adenosine deaminase, cause a severe developmental and epileptic encephalopathy. J. Med. Genet..

[34] Chan DCH (2021). Arhgef2 regulates mitotic spindle orientation in hematopoietic stem cells and is essential for productive hematopoiesis. Blood Adv..

[35] Koutelou E, Farria AT, Dent SYR (2021). Complex functions of *Gcn5* and *Pcaf* in development and disease. Biochim. Biophys. Acta Gene Regul. Mech..

[36] Oatley M (2020). Single-cell transcriptomics identifies CD44 as a marker and regulator of endothelial to haematopoietic transition. Nat. Commun..

[37] Qin P (2021). Integrated decoding hematopoiesis and leukemogenesis using single-cell sequencing and its medical implication. Cell Discov..

[38] Bailey TL, MacHanick P (2012). Inferring direct DNA binding from ChIP-seq. Nucleic Acids Res..

[39] Sizemore GM, Pitarresi JR, Balakrishnan S, Ostrowski MC (2017). The ETS family of oncogenic transcription factors in solid tumours. Nat. Rev. Cancer.

[40] Ran D (2013). RUNX1a enhances hematopoietic lineage commitment from human embryonic stem cells and inducible pluripotent stem cells. Blood.

[41] Ganly P, Walker LC, Morris CM (2004). Familial mutations of the transcription factor RUNX1 (AML1, CBFA2) predispose to acute myeloid leukemia. Leuk. Lymphoma.

[42] Cunningham L (2023). Natural history study of patients with familial platelet disorder with associated myeloid malignancy. Blood.

[43] Stockley J (2013). Enrichment of *FLI1* and *RUNX1* mutations in families with excessive bleeding and platelet dense granule secretion defects. Blood.

[44] Zerella JR (2023). Transcription factor genetics and biology in predisposition to bone marrow failure and hematological malignancy. Front. Oncol..

[45] Xiang P (2022). Elucidating the importance and regulation of key enhancers for human *MEIS1* expression. Leukemia.

[46] Wang C (2021). FLI1 Induces megakaryopoiesis gene expression through WAS/WIP-dependent and independent mechanisms; implications for Wiskott-Aldrich syndrome. Front. Immunol..

[47] Eckstein M, Rea M, Fondufe-Mittendorf YN (2017). Transient and permanent changes in DNA methylation patterns in inorganic arsenic-mediated epithelial-to-mesenchymal transition. Toxicol. Appl. Pharmacol..

[48] Wang Q (2022). Gene body methylation in cancer: Molecular mechanisms and clinical applications. Clin. Epigenet..

[49] Lange L, Morgan M, Schambach A (2021). The hemogenic endothelium: A critical source for the generation of PSC-derived hematopoietic stem and progenitor cells. Cell. Mol. Life Sci..

[50] Noetzli LJ, French SL, Machlus KR (2019). New insights into the differentiation of megakaryocytes from hematopoietic progenitors. Arterioscler. Thromb. Vasc. Biol..

[51] Martin M (2011). Cutadapt removes adapter sequences from high-throughput sequencing reads. EMBnet J..

[52] Schmieder R, Edwards R, Bateman A (2011). Quality control and preprocessing of metagenomic datasets. Bioinformatics.

[53] Li H, Durbin R (2009). Fast and accurate short read alignment with Burrows-Wheeler transform. Bioinformatics.

[54] Li H, Durbin R (2010). Fast and accurate long-read alignment with Burrows-Wheeler transform. Bioinformatics.

[55] Li, H. Aligning sequence reads, clone sequences and assembly contigs with BWA-MEM. 10.48550/arXiv.1303.3997 (2013).

[56] Li H (2009). The sequence alignment/map format and SAMtools. Bioinformatics.

[57] Cibulskis K (2013). Sensitive detection of somatic point mutations in impure and heterogeneous cancer samples. Nat. Biotechnol..

[58] Koboldt DC (2012). VarScan 2: Somatic mutation and copy number alteration discovery in cancer by exome sequencing. Genome Res..

[59] Ye K, Schulz MH, Long Q, Apweiler R, Ning Z (2009). Pindel: A pattern growth approach to detect break points of large deletions and medium sized insertions from paired-end short reads. Bioinformatics.

[60] Cingolani P (2012). Using *Drosophila melanogaster* as a model for genotoxic chemical mutational studies with a new program, SnpSift. Front. Genet..

[61] Quinlan AR, Hall IM (2010). BEDTools: A flexible suite of utilities for comparing genomic features. Bioinformatics.

[62] Bae S, Park J, Kim J-S (2014). Cas-OFFinder: A fast and versatile algorithm that searches for potential off-target sites of Cas9 RNA-guided endonucleases. Bioinformatics.

[63] Shen W, Le S, Li Y, Hu F (2016). SeqKit: A cross-platform and ultrafast toolkit for FASTA/Q file manipulation. PLoS One.

[64] Cantu VA, Sadural J, Edwards R (2019). PRINSEQ++, a multi-threaded tool for fast and efficient quality control and preprocessing of sequencing datasets. PeerJ. Prepr..

[65] Krueger F, Andrews SR (2011). Bismark: A flexible aligner and methylation caller for Bisulfite-Seq applications. Bioinformatics.

[66] Akalin A (2012). methylKit: a comprehensive R package for the analysis of genome-wide DNA methylation profiles. Genome Biol.

[67] Livak KJ, Schmittgen TD (2001). Analysis of relative gene expression data using real-time quantitative PCR and the 2^−ΔΔCT^ method. Methods.

[68] Langmead B, Salzberg SL (2012). Fast gapped-read alignment with Bowtie 2. Nat. Methods.

[69] Ramírez F (2016). deepTools2: A next generation web server for deep-sequencing data analysis. Nucleic Acids Res..

