# Supplementary material for: FLI1 is associated with regulation of DNA methylation and megakaryocytic differentiation in FPDMM caused by a RUNX1 transactivation domain mutation
Source: Sci Rep. 2024 Jun 18;14:14080. doi: 10.1038/s41598-024-64829-4 (PMC11189521; doi:10.1038/s41598-024-64829-4)

Supplementary Methods

Microarray analysis

The GSE54295 microarray data were downloaded from the ArrayExpress database (https://www.ebi.ac.uk/biostudies/arrayexpress/studies/E-GEOD-54295?query=GSE54295). Using the robust multi-array average algorithm from the oligo package[1], the raw data were background-corrected and normalized, and the probe-level to feature-level data were calculated. The dataset was annotated using the hugene20sttranscriptcluster.db package[2]. Differential gene expression analysis was performed using the limma package[3].

Single-cell RNA sequencing

Single-cell RNA sequencing libraries were constructed using the Chromium Single Cell 3' Reagent Kits v2 (10X Genomics, Pleasanton, CA, USA) according to the manufacturer's instructions. The prepared Illumina sequencer-adapted sequencing libraries were converted to a single-strand circular DNA libraries compatible with the MGI sequencing platform and sequenced using 150-bp paired-end reads on the DNBSEQ G400 (MGI). Preprocessing of single-cell RNA sequencing data, including quality control, normalization, batch effect correction, and cell cycle effect subtraction, was performed

according to the method of Yoshino, T. *et al*. (*Science*, 2021)[4]. Quality control cutoffs of more than 2500 detected counts per cell and less than 8000 and a ratio of mitochondrial counts per cell of less than 12.5% were used. Clustering and dimensionality reduction were performed according to the method of Yoshino, T. *et al*.[4] using the PCA spaces from PC 1 to PC 13 with resolution = 0.2 for all cell analyses. Identification of cluster-specific genes and their visualization was performed according to the method of Yoshino, T. *et al*.[4] with the parameters min.pct = 0.25 and logfc.threshold = 0.25 and with the MAST algorithm[5] using FindAllMarkers implemented in the Seurat package.

References

1. Carvalho, B. S. & Irizarry, R. A. A framework for oligonucleotide microarray preprocessing. *Bioinformatics* **26**, 2363–2367 (2010).
2. MacDonald, J. W. hugene20sttranscriptcluster.db: Affymetrix hugene20 annotation data (chip hugene20sttranscriptcluster). R package version 8.7.0. *Bioconductor* https://doi.org/doi:10.18129/B9.bioc.hugene20sttranscriptcluster.db (2017).
3. Ritchie, M. E. *et al*. limma powers differential expression analyses for RNA-sequencing and microarray studies. *Nucleic Acids Res* **43**, e47 (2015).
4. Yoshino T. *et al*. Generation of ovarian follicles from mouse pluripotent stem cells. *Science* **373**, eabe0237 (2021).
5. Finak G. *et al*. MAST: a flexible statistical framework for assessing transcriptional changes and characterizing heterogeneity in single-cell RNA sequencing data. *Genome Biol* **16**, 278 (2015).

Supplementary Figure Legends

Supplementary Figure 1 | Evaluation of genome editing efficiencies and off-target effects.

(a) Agarose gel electrophoresis of PCR products after T7E1 reaction. WT refers to untreated wild-type human iPSCs as a negative-control. RD refers to cells into which the RNP complex and donor DNA were delivered by electroporation. Left and right panels indicate the RUNX1$^{WT/R201Q}$ and RUNX1$^{WT/Y287X}$ mutation mimics, respectively. In the T7E1 assay, PCR was followed by denaturation, renaturing, and enzymatic treatment. If a mutation was present, a mismatched fragment was formed, which was cleaved by T7E1. The wild-type control cells showed only 1205 bp fragments, which were consistent with the expected PCR product size (WT of left panel). However, cells that were electroporated with R201Q-specific genome editing machinery showed two shorter fragments of approximately 660 and 550 bp in addition to the 1205 bp fragments (RD of left panel). Similarly, cells electroporated with Y287X-specific genome editing machinery also showed two shorter fragments (approximately 490 and 370 bp) in addition to the fragments matching the expected PCR product size (860 bp) (RD of right panel), whereas wild-type cells showed only 860 bp fragments (WT of right panel).

(b) Overlap of genomic sites between predicted off-target candidate sites for

crRNAs (blue for RUNX1^{R201Q}-crRNA and red for RUNX1^{Y287X}-crRNA) and putative SNVs detected by two variant callers, MuTect2 (dark blue) and VarScan2 (yellow), in RUNX1$^{WT/R201Q}$ (left) and RUNX1$^{WT/Y287X}$ iPSCs (right). The 491 and 397 off-target candidate sites containing mismatches of up to 5 bases with RUNX1^{R201Q}-crRNA and RUNX1^{Y287X}-crRNA, respectively, were identified. No putative SNVs were identified at the predicted off-target candidate sites.

(c) Overlaps of genomic sites between predicted off-target candidate sites for crRNAs (blue for RUNX1^{R201Q}-crRNA and red for RUNX1^{Y287X}-crRNA) and putative insertion/deletion (indels) detected by three variant callers, MuTect2 (dark blue), VarScan2 (yellow), and Pindel (gray), in RUNX1$^{WT/R201Q}$ (left) and RUNX1$^{WT/Y287X}$ iPSCs (right). No putative indels were identified at the predicted off-target candidate sites.

(d) A table of all the putative SNVs at protein-coding regions validated by WES analysis. Shown left to right is the mutant iPSC line affected by the SNV, the name of gene affected by the SNV, the genomic location of the SNV, and the pattern of transversion mutation.

(e) Bulk sequence traces of wild-type (left), RUNX1$^{WT/R201Q}$ (middle), and RUNX1$^{WT/Y287X}$ iPSCs (right) around the genomic regions where the putative SNVs validated by WES analysis are located. Stars (★) represent putative SNVs. Of the eight

putative SNVs, three were specifically identified in RUNX1$^{WT/R201Q}$ iPSCs and were located in the *ADARB1*, *CNGB3*, and *NAALADL1* loci. Additionally, five were specifically identified in RUNX1$^{WT/Y287X}$ iPSCs and were located in the *ARHGEF2*, *GRM6*, *KAT2B*, *TAF5L*, and *ZFHX3* loci.

(f) Confirmation of the expression of *ADARB1*, *ARHGEF2*, *KAT2B*, and *TAF5L* in wild-type HPCs (dark gray) compared with wild-type iPSCs (light gray) by qRT-PCR. The *X*-axis indicates the target genes, and the *Y*-axis indicates the fold-change. Data are presented as the mean ± SD of three biological replicates. Asterisks denote significant difference: *$P < 0.05$ and ns, not significant. Of the eight validated SNVs, four genes affected by SNVs (*CNGB3*, *GRM6*, *NAALADL1*, and *ZFHX3*) were not expressed in HPCs, whereas one gene affected by the SNV (*TAF5L*) was expressed at the same levels in wild-type iPSCs and HPCs, suggesting that those SNVs are irrelevant. The remaining three SNVs affected *ADARB1*, *ARHGEF2*, and *KAT2B*. The expression of these genes showed an upregulation tendency in HPCs compared with iPSCs.

Supplementary Figure 2 | Evaluation of *in vitro* megakaryocytic differentiation processes of FPDMM-mimicking iPSCs.

(a) Representative images of wild-type (upper left), RUNX1$^{WT/R201Q}$ (R201Q, upper

right), and RUNX1$^{WT/Y287X}$ (Y287X, lower left) cells before passaging iPSCs (upper left of each group image) and on Day 1, 3, 5, 7, and 12 of *in vitro* hematopoietic differentiation using STEMdiff^{TM} Hematopoietic Kit.

(b) A table of average absolute numbers of CD34$^+$CD45$^+$ HPCs in wild-type (WT), RUNX1$^{WT/R201Q}$ (R201Q), and RUNX1$^{WT/Y287X}$ (Y287X) iPSC samples per well of a 24-well plate.

(c) Confirmation of a potential bias in a cell state underlying wild-type and FPDMM-mimicking RUNX1$^{WT/R201Q}$ HPCs by single-cell RNA sequencing analysis. Wild-type (right) and FPDMM-mimicking RUNX1$^{WT/R201Q}$ (abbreviated as R201Q; left) HPCs were dimension-reduced and visualized using a uniform manifold approximation and projection (UMAP) algorithm. As a result, these cells could be divided into three subclusters. Subclusters were colored red (cluster 0), green (cluster 1), or blue (cluster 2), respectively.

(d) Heatmap of the top ten marker genes for each subcluster. The color ranges from pink to yellow indicate the normalized and log-transformed expression levels from low to high, respectively. Based on the expression of known hematopoietic cell-type-specific genes, they were mapped into three cell types: erythroid lineage (cluster 0), early hematopoietic progenitors (clusters 1), and megakaryoid lineage (clusters 2).

(e) Stacked bar plot of cell type proportions in wild-type and RUNX1$^{WT/R201Q}$ HPCs. *X*-axis indicates the sample names, and *Y*-axis indicates the percentage of each subcluster in each sample (percent clusters). These results show that cluster 0 comprises the majority of FPDMM-mimicking RUNX1$^{WT/R201Q}$ HPCs compared to the wild-type cells (81.1% vs. 51.9%), with a decreasing percentage of clusters 1 (9.8% vs. 24.7%) and 2 (9.1% vs. 23.4%).

(f) Representative images of wild-type (left), RUNX1$^{WT/R201Q}$ (R201Q, middle), and RUNX1$^{WT/Y287X}$ (Y287X, right) cells on Day 15 of *in vitro* megakaryocytic differentiation.

(g) A table of percentages of CD41^{+}CD42b^{+} Mks per 20 000 of these HPCs.

Supplementary Figure 3 | Enrichment analysis of TF-binding motifs and expression analysis of ETS family TFs in FPDMM-mimicking HPCs.

(a) Enrichment distributions of ETS family TF-binding motifs. Solid lines represent probabilities at ± 5 kb from the commonly hypermethylated DMCs between RUNX1$^{WT/R201Q}$ and RUNX1$^{WT/Y287X}$ HPCs, and dashed lines represent probabilities at ± 5 kb from randomly selected CpGs. Red: GABPA-binding motif, light green: FEV-binding motif, orange: ELF1-binding motif, blue: ELF2-binding motif, and pink: ELK1-

binding motif.

(b) Distribution of ETS family TF (FEV)-binding motif enrichment. The solid line represents the probability at ± 5 kb from RUNX1$^{WT/Y287X}$ HPC-specific hypermethylated DMCs, and the dashed line represents the probability at ± 5 kb from randomly selected CpGs.

(c) Distributions of RUNX1-binding motif enrichment. Solid lines represent the probabilities at ± 5 kb for hypermethylated DMCs in RUNX1$^{WT/R201Q}$ (upper left) and RUNX1$^{WT/Y287X}$ (upper right) HPCs, and hypomethylated DMCs in RUNX1$^{WT/R201Q}$ (lower left) and RUNX1$^{WT/Y287X}$ (lower right) HPCs. Dashed lines represent the probabilities at ± 5 kb from randomly selected CpGs.

(d) Confirmation of the expression of 16 ETS family TFs in RUNX1$^{WT/R201Q}$ (blue) and RUNX1$^{WT/Y287X}$ HPCs (red) compared with that in wild-type cells by qRT-PCR. The *X*-axis indicates the target genes, and the *Y*-axis indicates the fold-change. Data are presented as the mean ± SD of four biological replicates. Asterisks denote significant difference: $*P < 0.05$ and $**P < 0.01$. The expression of 12 of 28 ETS family TFs (*EHF*, *ELF3*, *ELF5*, *ETV1*, *ETV2*, *ETV3L*, *ETV4*, *ETV7*, *FEV*, *SPIB*, *SPIC*, and *SPDEF*) was undetermined.

(e) Confirmation of the expression of *ELF1* and *FLI1* in cells hematopoietically

differentiated from patient-derived iPSCs equivalent to RUNX1$^{WT/Y287X}$. The comparison was performed using two mutation-corrected FPDMM clones (isogenic controls clone E and clone F) by microarray analysis. The *X*-axis indicates the target genes, and the *Y*-axis indicates the fold-change. Data are presented as the mean ± SD of three biological replicates. The expression of *FLI1* tended to be lower in patient-derived cells than in isogenic control clone E-derived cells (Y287X-equivalent vs. isogenic control clone E; *P* = 0.049, false discovery rate adjusted by the Benjamini-Hochberg method [FDR] = 0.18), as well as in patient-derived cells than in isogenic control clone F-derived cells (Y287X-equivalent vs. isogenic control clone F; *P* = 0.014, FDR = 0.19). Similarly, the expression of *ELF1* tended to be lower in patient-derived cells than in isogenic control clone E-derived cells (Y287X-equivalent vs. isogenic control clone E; *P* = 0.052, FDR = 0.18), as well as in patient-derived cells than in isogenic control clone F-derived cells (Y287X-equivalent vs. isogenic control clone F; *P* = 0.077, FDR = 0.38).

(f) (Left) The known HOCOMOCO v11 binding motif for FLI1 (top) and ETV6 (bottom). S: G/C, R: A/G, and M: A/C. (Right) Distribution of FLI1- and ETV6-binding motif-enrichment. The *X*- and *Y*-axes show the distance from DMC (bp) and probability of TF-binding motifs, respectively. Solid lines are probabilities at ± 5 kb for hypermethylated DMCs in RUNX1$^{WT/Y287X}$ HPCs, and dashed lines are probabilities at ±

5 kb from randomly selected CpGs. Purple: FLI1-binding motif and orange: ETV6-binding motif.

Supplementary Figure 4 | Overlap of demethylated CpGs in *FLI1*-overexpressing iPSCs and hypermethylated CpGs between RUNX1$^{WT/R201Q}$ and RUNX1$^{WT/Y287X}$ HPCs.

The "demeth_FLI1-iPSC", "hypermeth_R201Q", and "hypermethy_Y287X" refer to demethylated CpGs in *FLI1*-overexpressing iPSCs, hypermethylated CpGs between RUNX1$^{WT/R201Q}$ HPCs, and hypermethylated CpGs between RUNX1$^{WT/Y287X}$ HPCs, respectively.

Supplementary Figure 5 | Evaluation of *in vitro* megakaryocytic differentiation efficiencies of *FLI1*-knockdown iPSCs.

(a) Average absolute numbers of CD34^{+}CD45^{+} HPCs in negative-control-knockdown (nc-KD, gray) and *FLI1*-knockdown (FLI1-KD, yellow) iPSC samples per well of a 24-well plate. Data are presented as the mean ± SD of three biological replicates.

(b) A table of average absolute numbers of CD34^{+}CD45^{+} HPCs in negative-control-knockdown (nc-KD) and *FLI1*-knockdown (FLI1-KD) iPSC samples per well of a 24-

well plate and percentages of CD41^{+}CD42b^{+} Mks per 20 000 of these HPCs.

Supplementary Figure 6 | Evaluation of hematopoietic differentiation efficiencies in FPDMM-mimicking HPCs overexpressing *FLI1*.

(a) Average absolute numbers of CD34^{+}CD45^{+} HPCs in wild-type (WT, gray), RUNX1$^{WT/Y287X}$-mock-control (Y287X-mock, red), and RUNX1$^{WT/Y287X}$-FLI1-overexpressing (Y287X-FLI1, dark orange) iPSC samples per well of a 24-well plate. Data are presented as the mean ± SD of six biological replicates. Asterisks denote significant difference: *$P < 0.05$ and ns, not significant.

(b) Representative plot for flow cytometric analysis of CD41^{+}CD42b^{+} Mks per 20 000 wild-type (upper left), Y287X-mock (lower left), and Y287X-FLI1 (lower right) HPCs.

(c) A table of average absolute numbers of CD34^{+}CD45^{+} HPCs in wild-type (WT), Y287X-mock, and Y287X-FLI1 iPSC samples per well of a 24-well plate and percentages of CD41^{+}CD42b^{+} Mks per 20 000 of these HPCs.

(d) Confirmation of the expression of *FLI1* in R201Q-mock (blue) and R201Q-FLI1 (dark blue) HPCs using qRT-PCR. Data are presented as the mean ± SD of six biological replicates. The ns denotes not significant.

(e) Percentages of $CD41^{+}CD42b^{+}$ Mks per 20 000 wild-type (WT, gray), R201Q-mock (blue), and R201Q-FLI1 (dark blue) HPCs. Data are presented as the mean ± SD of six biological replicates. The ns denotes not significant.

(f) A table of average absolute numbers of $CD34^{+}CD45^{+}$ HPCs in R201Q-mock and R201Q-FLI1 iPSC samples per well of a 24-well plate and percentages of $CD41^{+}CD42b^{+}$ Mks per 20 000 of these HPCs.

Supplementary Figure 7 | Evaluation of mean methylation percentage points in FPDMM-mimicking HPCs overexpressing *FLI1*.

(a) Integrative genomics viewer screenshot showing the methylation ratios per sequencing coverages. The tracks included from top to bottom: DMC sites (within chr1:111,204,290-111,204,976) that were hypermethylated in $RUNX1^{WT/Y287X}$ HPCs compared with wild-type cells; sequencing coverage of Y287X-mock HPCs; sequencing reads of Y287X-mock HPCs; sequencing coverage of Y287X-FLI1 HPCs; sequencing reads of Y287X-FLI1 HPCs; genes. In the coverage and read tracks, the methylated and unmethylated CpG sites are highlighted in red and blue, respectively.

(b) Confirmation of the mean percentage points of each CpG group between Y287X-FLI1 and Y287X-mock HPCs. FLI1-mock refers to the mean percentage point of 1344

CpG sites between Y287X-FLI1 and Y287X-mock HPCs, whereas random refers to that of 1344 CpG sites randomly selected five times among CpGs covered by sequencing. The asterisk denotes significant difference: ***$P < 0.001$. The mean percentage point was significantly lower in FLI1-mock compared with that in random ($P = 0.0001$, two sample *t*-test).

Supplementary Table Legends

Supplementary Table 1. Methylation percentage point differences between Y287X-FLI1 and Y287X-mock HPCs

Shown left to right is the genomic location (the name of the chromosome, the starting position in the chromosome, and the ending position in the chromosome) of the DMCs that were hypermethylated in RUNX1$^{WT/Y287X}$ HPCs compared with wild-type cells, the percent methylation score of Y287X-FLI1 HPCs at these sites, the percent methylation score of Y287X-mock HPCs at these sites, and the methylation percentage point difference between Y287X-FLI1 and Y287X-mock HPCs. Red color represents positive percentage points, and blue color represents negative percentage points in the "Percentage point" column.

Supplementary Table 2. crRNA and donor DNA list for CRISPR–Cas9

Sequences of crRNAs and donor DNAs. For each target *RUNX1* mutation, the crRNA and donor DNA are shown in the upper and lower row, respectively. The underlined area in the donor DNA box represents the knocked-in single-nucleotide mutation in *RUNX1*.

Supplementary Table 3. Primer list for T7E1 assay

Supplementary Table 4. Primer list for nested PCR

Supplementary Table 5. Primer list for SNV analysis

Supplementary Table 6. Primer list for qRT-PCR

Supplementary Figure. 1

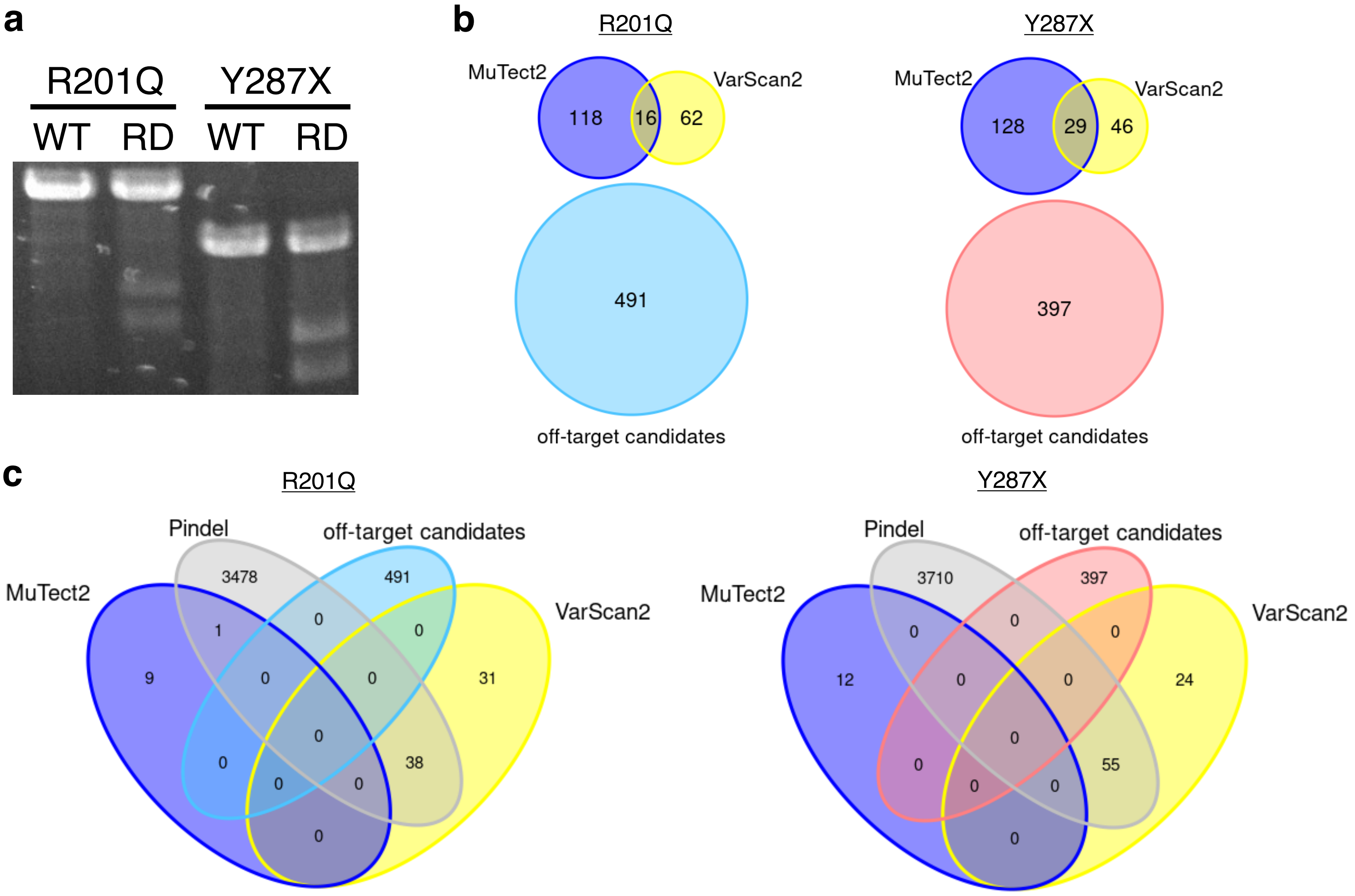

d

iPSC line	Affected gene	Genomic location	Transversion
R201Q	*ADARB1*	chr21:45176638	G > A
	CNGB3	chr8:86576124	G > T
	NAALADL1	chr11:65045892	A > G
Y287X	*ARHGEF2*	chr1:155965686	G > A
	GRM6	chr5:178986185	C > A
	KAT2B	chr3:20040638	C > T
	TAF5L	chr1:229594728	C > T
	ZFHX3	chr16:72795180	G > A

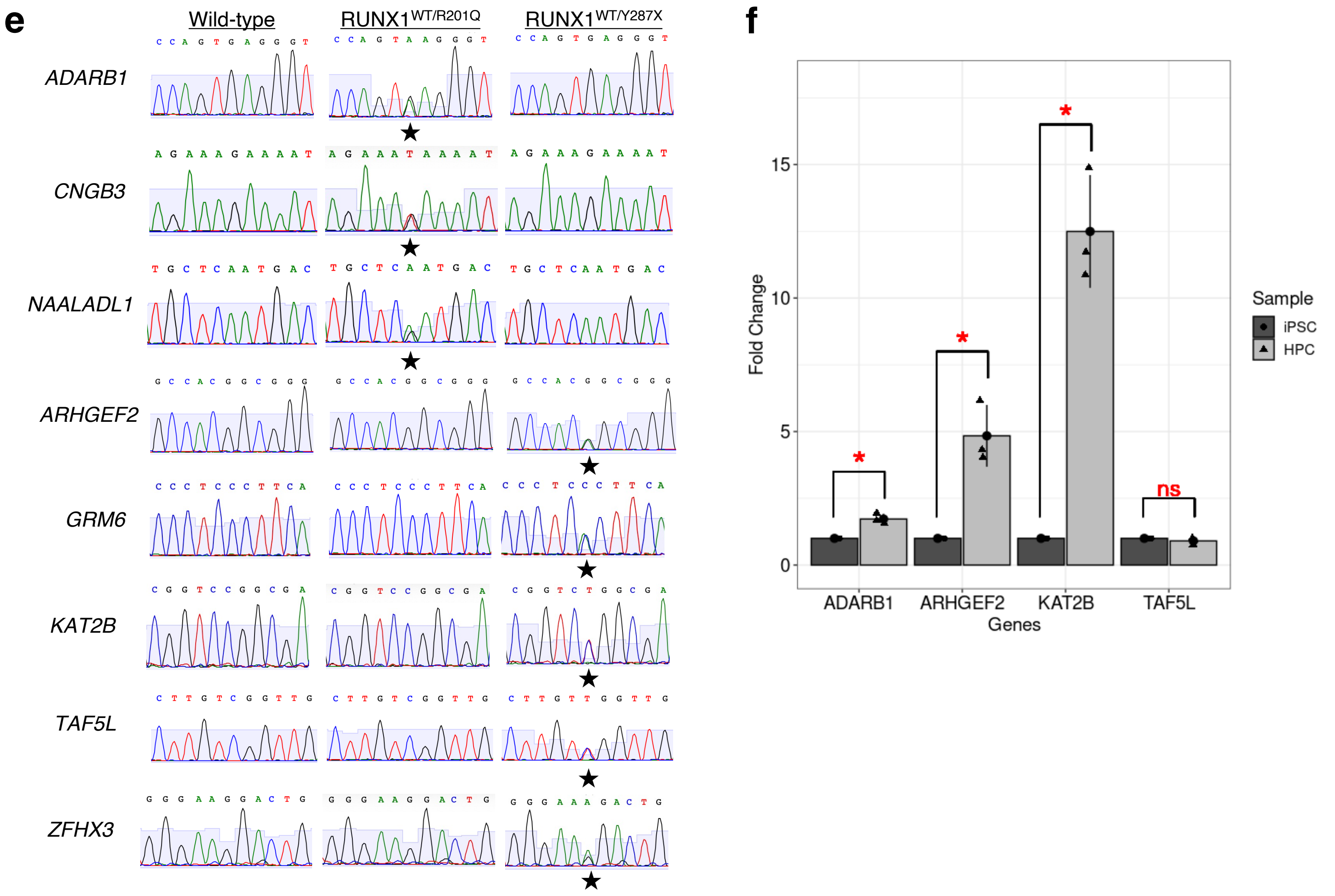

Supplementary Figure. 2

a

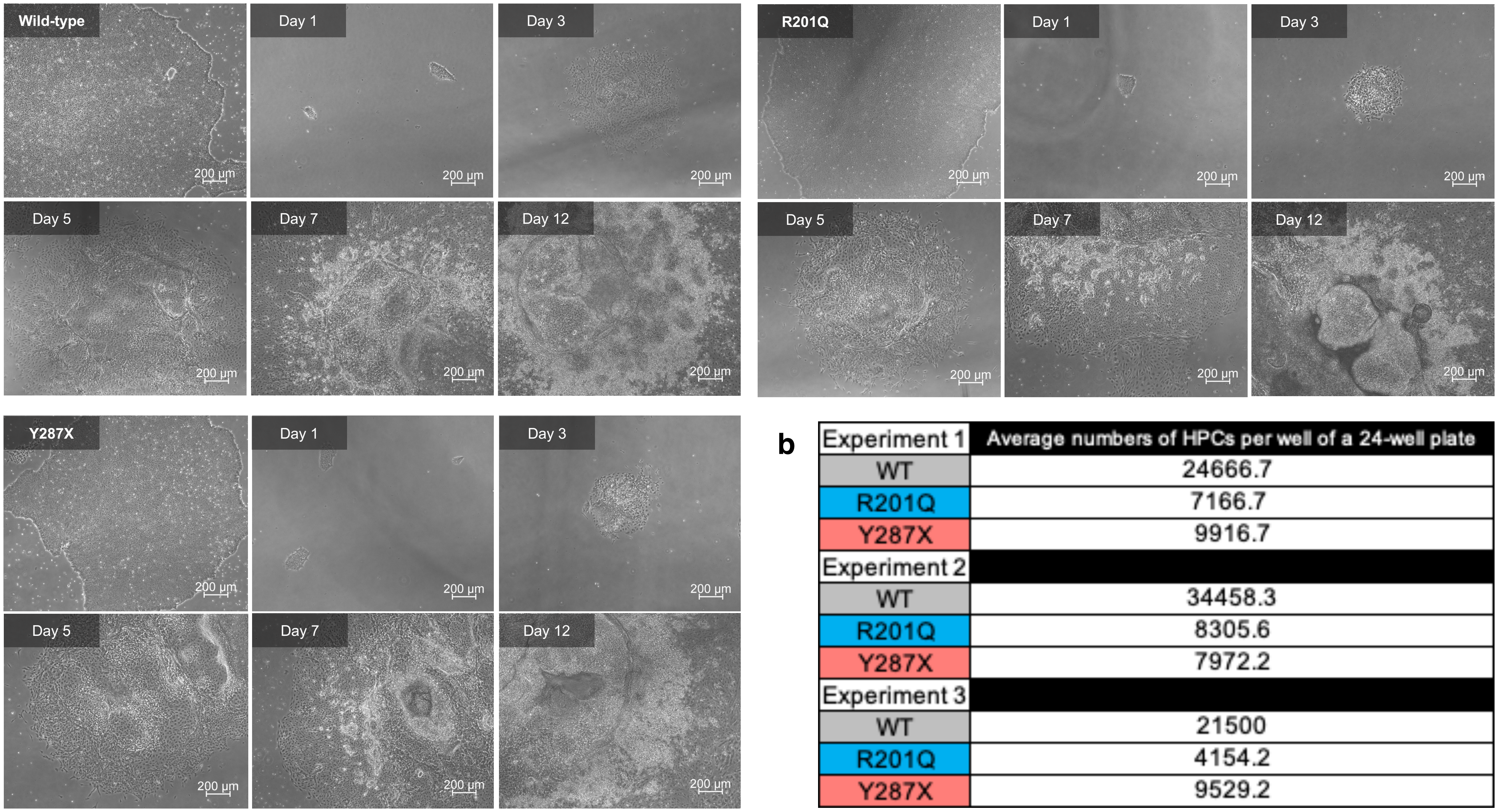

b

Experiment 1	Average numbers of HPCs per well of a 24-well plate
WT	24666.7
R201Q	7166.7
Y287X	9916.7
Experiment 2	
WT	34458.3
R201Q	8305.6
Y287X	7972.2
Experiment 3	
WT	21500
R201Q	4154.2
Y287X	9529.2

c

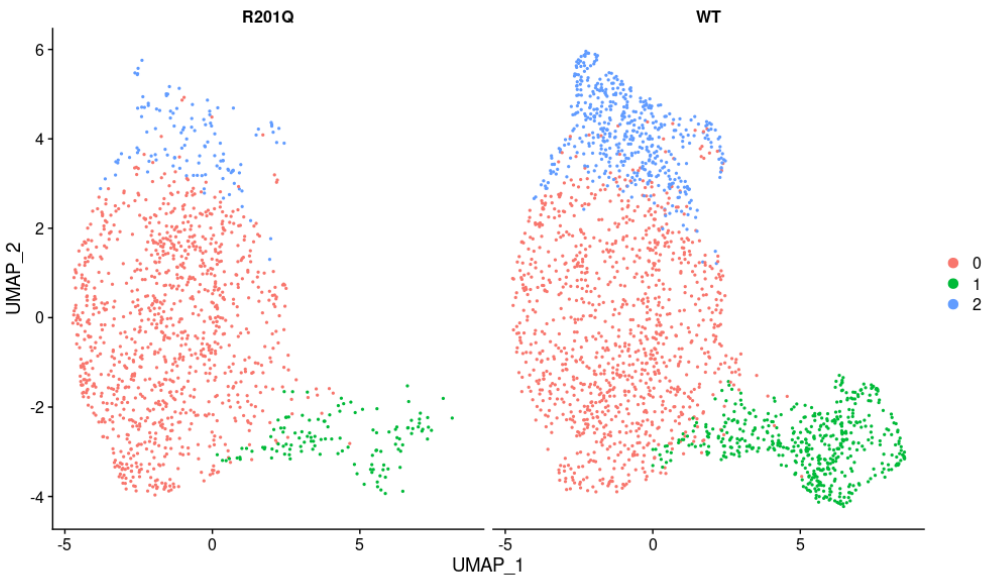

d

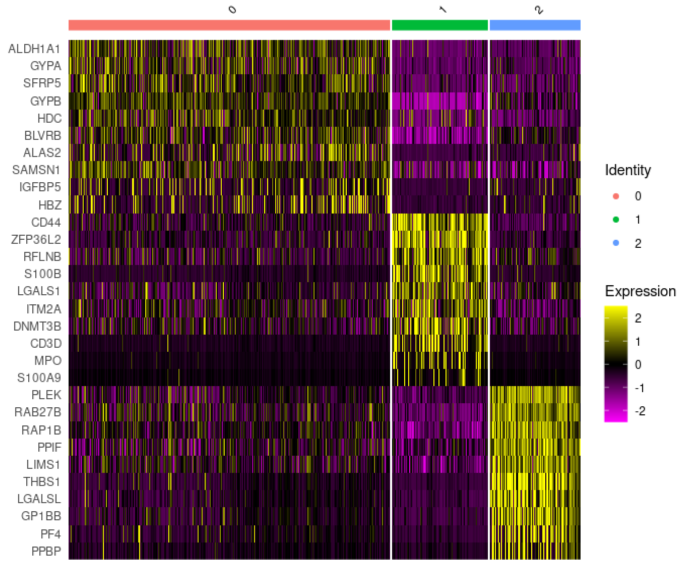

e

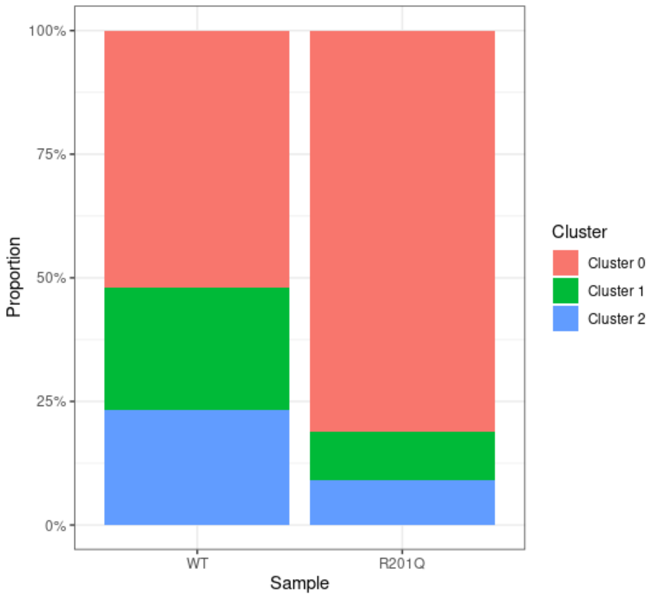

f

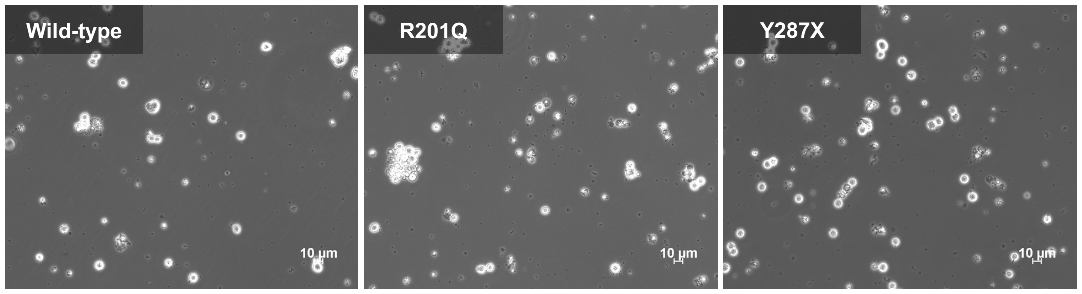

g

Experiment 1	Percentages of Mks
WT	62.9
R201Q	39.4
Y287X	43.5
Experiment 2	
WT	59.9
R201Q	29.3
Y287X	50.7
Experiment 3	
WT	62.1
R201Q	42.7
Y287X	50.7

Supplementary Figure. 3

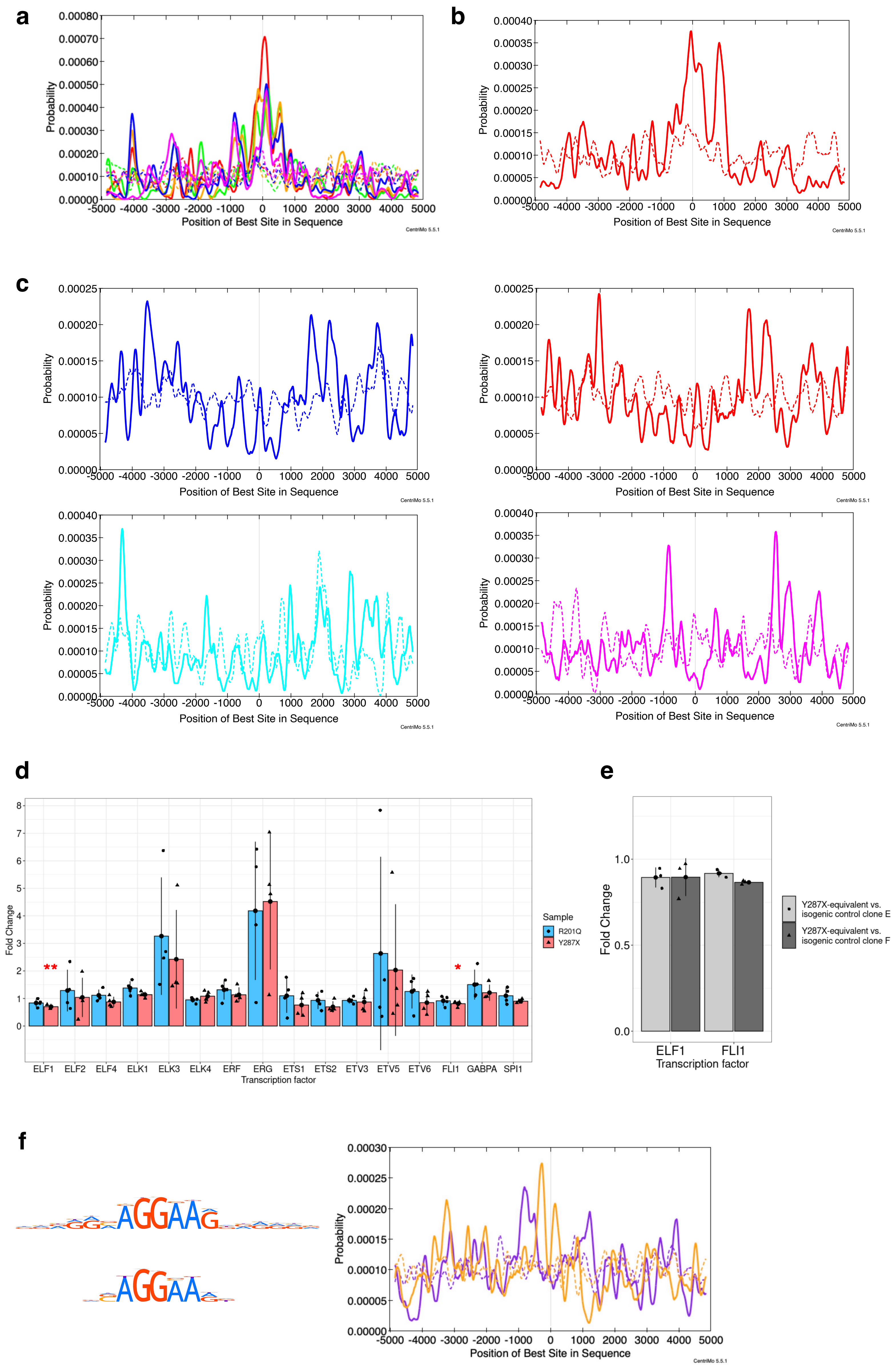

Supplementary Figure. 4

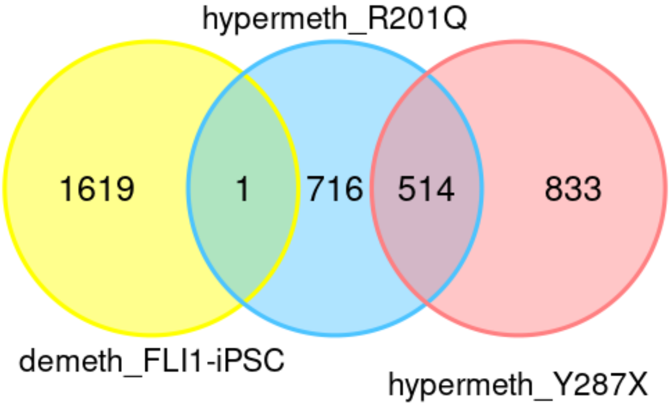

Supplementary Figure. 5

a

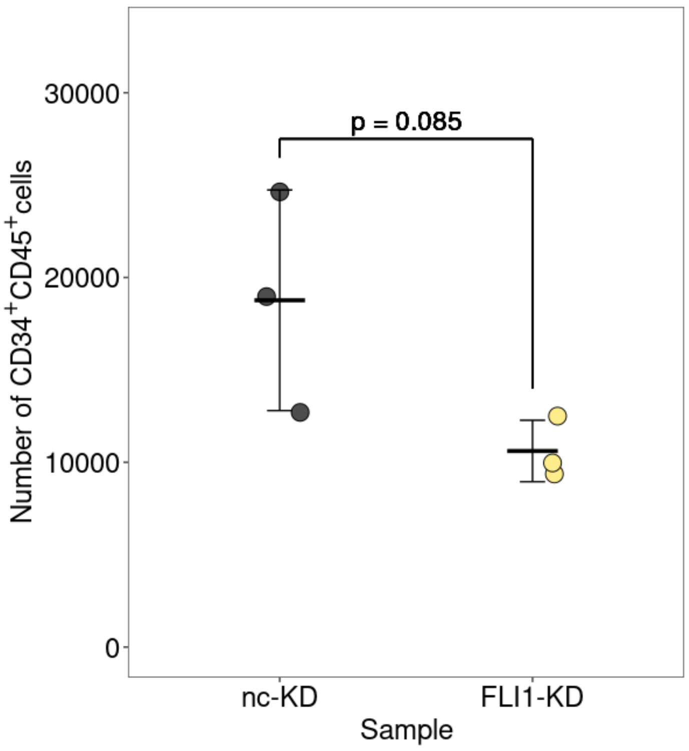

b

Experiment 1	Average numbers of HPCs per well of a 24-well plate	Percentages of Mks
FLI1-KD	9366.6	63.8
nc-KD	18966.6	69.2
Experiment 2		
FLI1-KD	12500	68.3
nc-KD	24633.3	71.8
Experiment 3		
FLI1-KD	9966.7	64.9
nc-KD	12700	74

Supplementary Figure. 6

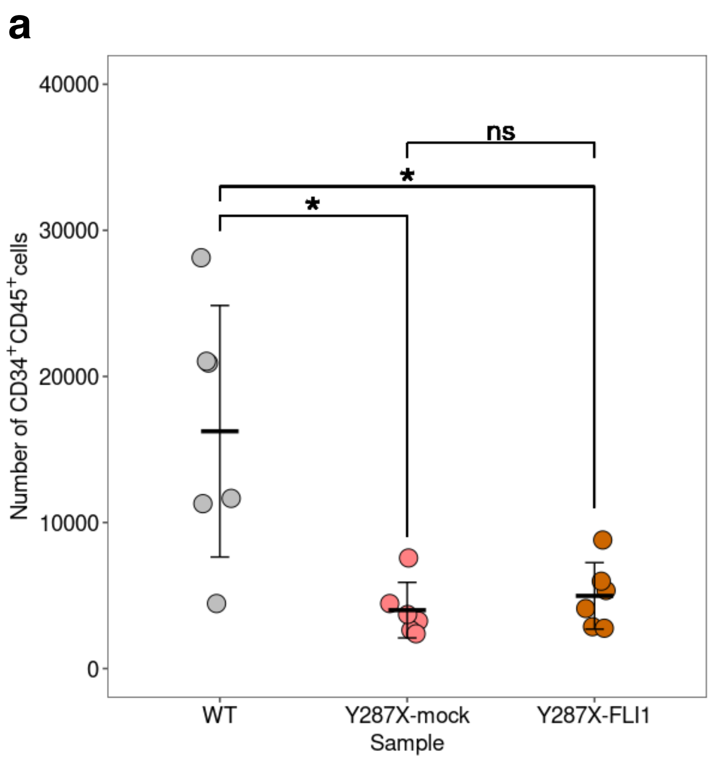

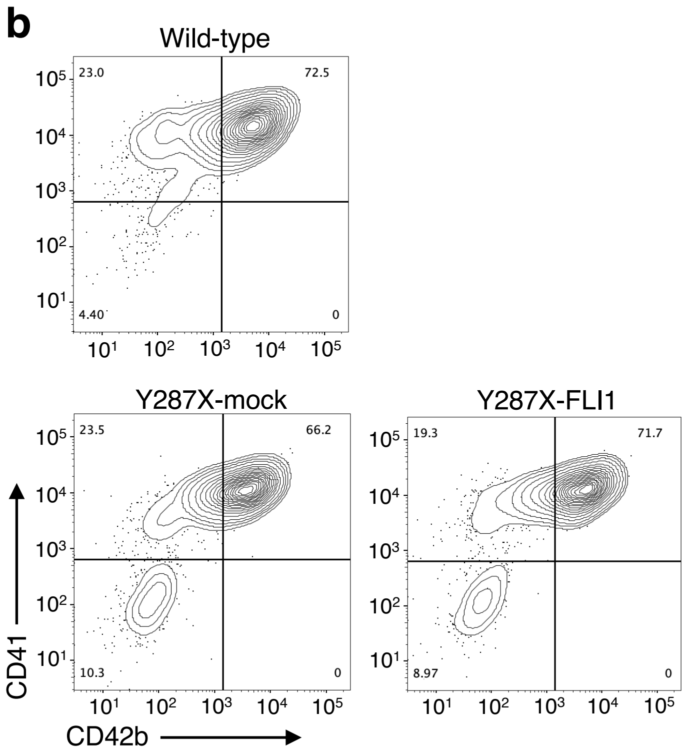

c

Experiment 1	Average numbers of HPCs per well of a 24-well plate	Percentages of Mks
WT	20916.7	69
Y287X-mock	3255.6	55.3
Y287X-FLI1	2863.3	57.3
Experiment 2		
WT	4450	75
Y287X-mock	2630	62.3
Y287X-FLI1	4110	65.7
Experiment 3		
WT	11287.5	62.3
Y287X-mock	3700	58.25
Y287X-FLI1	5340	60.43
Experiment 4		
WT	21037.5	58
Y287X-mock	4450	56.2
Y287X-FLI1	5980	56.7
Experiment 5		
WT	28125	72.5
Y287X-mock	7570	66.2
Y287X-FLI1	8800	71.7
Experiment 6		
WT	11650	51.3
Y287X-mock	2381	41.1
Y287X-FLI1	2760	44.2

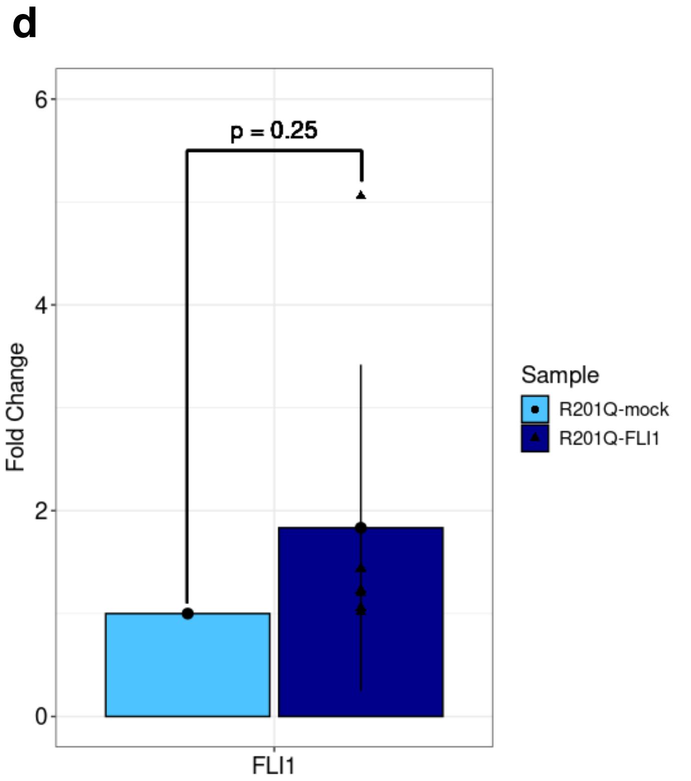

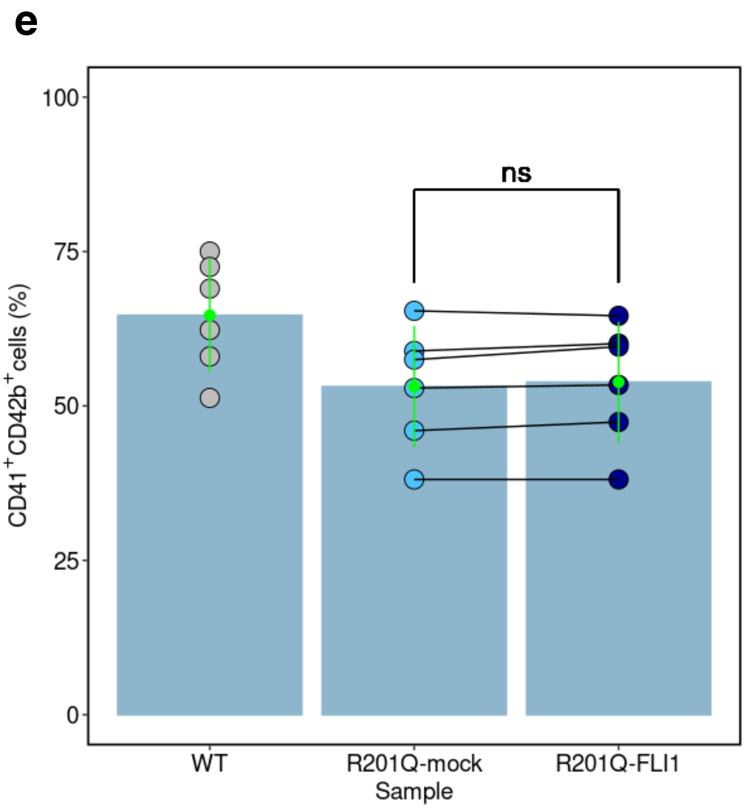

f

Experiment 1	Average numbers of HPCs per well of a 24-well plate	Percentages of Mks
R201Q-mock	4622.2	52.9
R201Q-FLI1	4955.6	53.4
Experiment 2		
R201Q-mock	6800	65.4
R201Q-FLI1	2870	64.6
Experiment 3		
R201Q-mock	4690	58.9
R201Q-FLI1	3990	60.1
Experiment 4		
R201Q-mock	9270	46
R201Q-FLI1	7210	47.4
Experiment 5		
R201Q-mock	10262.5	57.5
R201Q-FLI1	6962.5	59.6
Experiment 6		
R201Q-mock	9337.5	38.1
R201Q-FLI1	5575	38.1

Supplementary Figure. 7

a

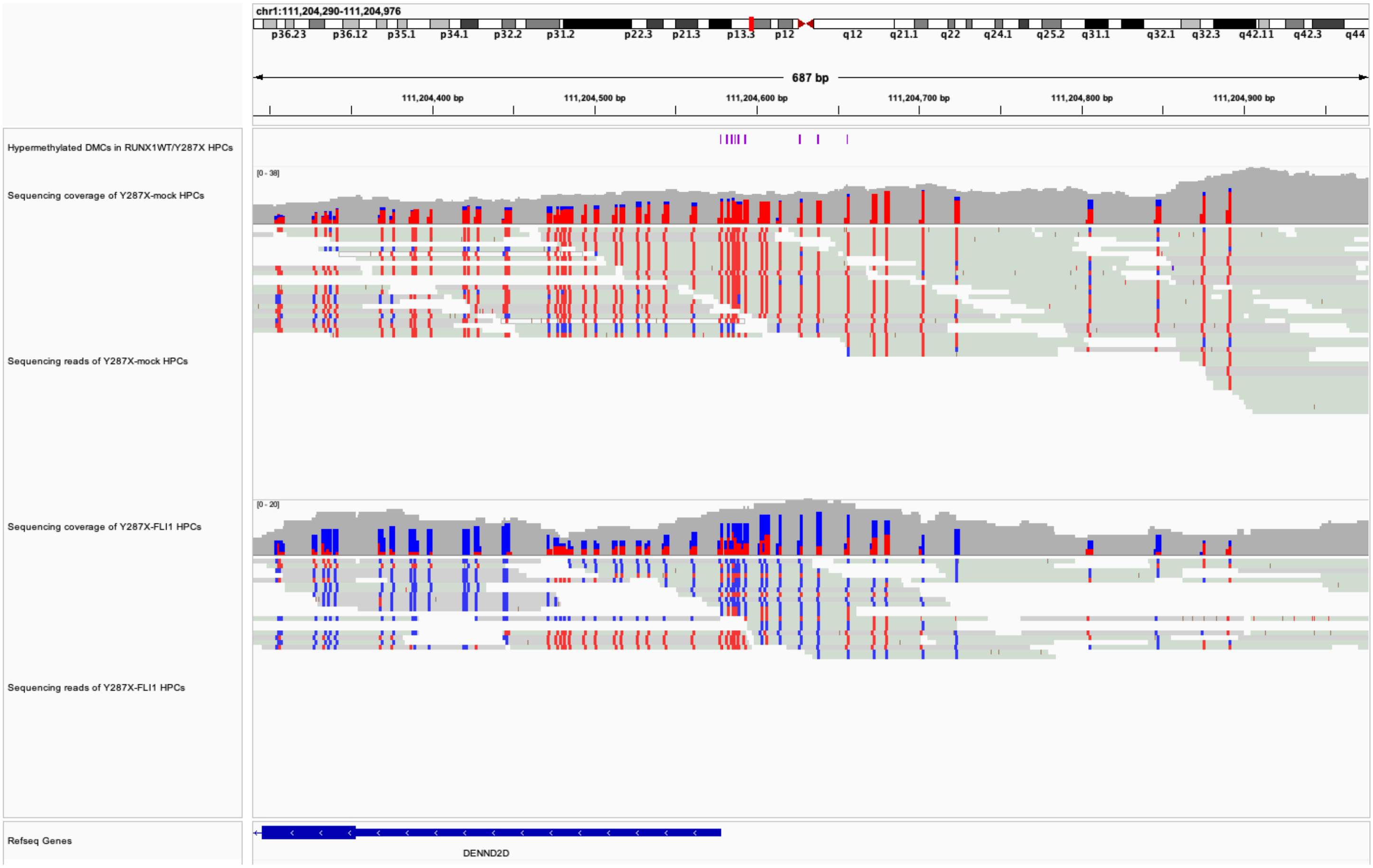

b

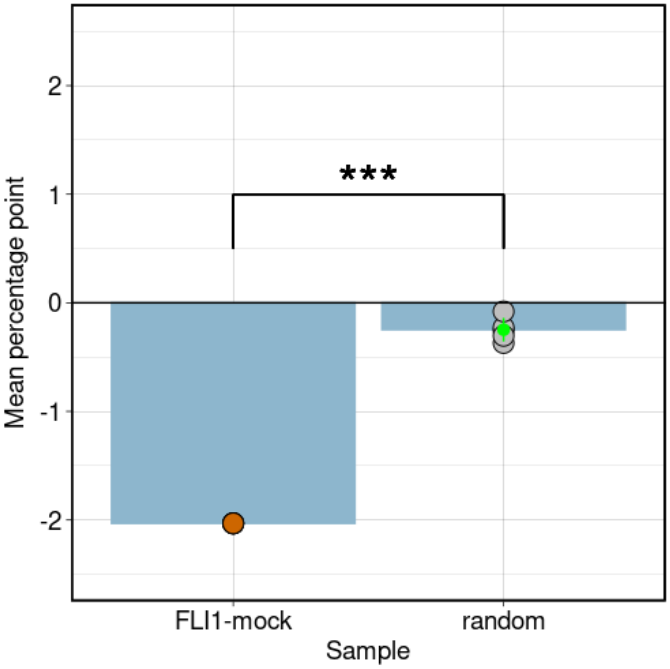

Supplement: Supplementary file 1 — Supplementary Information. [file 41598_2024_64829_MOESM1_ESM.pdf]
